# Cranial Appendages in Ruminants: Diversity, Evolution, Development, and Molecular Basis of Horns, Pronghorns, Antlers, and Ossicones

**DOI:** 10.3390/biology15141210

**Published:** 2026-07-22

**Authors:** Rafal P. Piprek, Izabela Rams-Pociecha, Paulina C. Mizia

**Affiliations:** 1 Department of Comparative Anatomy, Institute of Zoology and Biomedical Research, Faculty of Biology, Jagiellonian University, Gronostajowa 9, 30-387 Krakow, Poland; 2 Doctoral School of Exact and Natural Sciences, Jagiellonian University, 30-348 Krakow, Poland

**Keywords:** ruminants, cranial appendages, horns, antlers, pronghorns, ossicones, evolution, neural crest cells, *RXFP2*, regeneration, paleontology

## Abstract

Ruminants are unique among living mammals in possessing paired bony cranial appendages in the form of horns, pronghorns, antlers, or ossicones. This review summarizes current knowledge on the structure, development, function, and evolution of these appendages and integrates recent findings from genomics, transcriptomics, and single-cell analyses. Two competing hypotheses have been proposed to explain their origin. According to the independent-origin hypothesis, horns, pronghorns, antlers, and ossicones evolved separately in different pecoran lineages, possibly through repeated recruitment of similar developmental capacities of the frontal region of the skull. According to the common-origin hypothesis, these appendages derive from a single ancestral osseous cranial structure that was subsequently modified in different lineages. Comparative anatomy and developmental data reveal major differences among the four types of appendages, including dermally ossifying horn cores in bovids, annually regenerated antlers arising from frontal bone pedicles in cervids, deciduous keratin sheaths in pronghorns, and skin-covered ossicones in giraffids. The fossil record does not currently resolve these hypotheses, because the main appendage types are first documented within a relatively short early Miocene interval and transitional forms remain unknown. In contrast, molecular and cellular studies identify shared developmental pathways, cranial neural crest-derived progenitors, and conserved regulatory genes, including *RXFP2*, *ALX1*, *SOX9*, and components of Wnt signaling, which are consistent with a shared developmental module. We conclude that the homology of pecoran cranial appendages remains unresolved and that further paleontological, developmental, and comparative molecular studies are required to determine whether these structures share a common evolutionary origin or instead represent convergent recruitment of similar developmental programs.

## Simple Summary

Ruminants, including cattle, antelopes, pronghorns, deer and giraffes, are the only living mammals with cranial appendages with a bony component such as horns, pronghorns, antlers, and ossicones. Despite their diverse forms and growth patterns, these structures may share a common evolutionary origin, although an alternative hypothesis proposes that they evolved independently in different ruminant lineages. This review evaluates anatomical, developmental, paleontological, phylogenetic, and molecular evidence bearing on both possibilities. Understanding ruminant cranial appendages provides insights into mammalian evolution and offers valuable perspectives for regenerative biology and medicine.

## 1. Introduction

**Figure 1:**
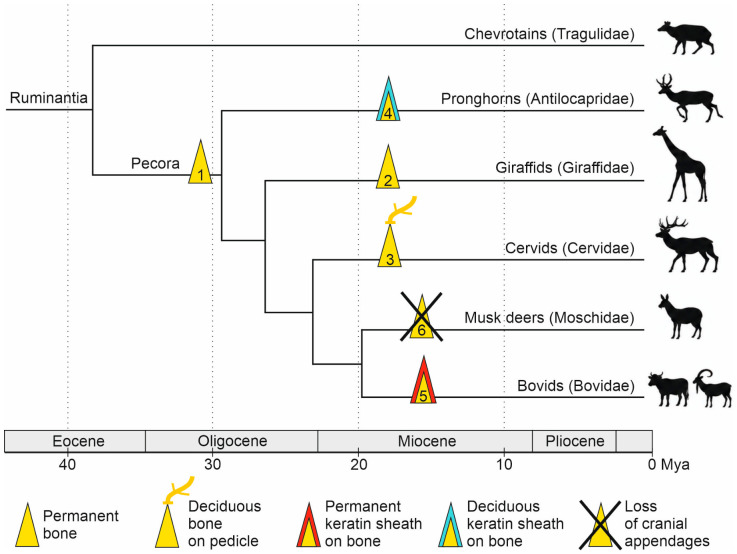
Phylogenetic tree illustrating relationships among the families of Ruminantia. The lineage leading to Tragulidae diverged from the remaining ruminants during the Eocene, before the evolution of cranial appendages. Osseous cranial appendages (1) probably originated in the late Oligocene or early Miocene, before or during the early diversification of Pecora. Different types of cranial appendages subsequently evolved within Pecora. Giraffids (2) and cervids (3) possess mature cranial appendages composed primarily of bone and lacking a keratinous sheath. In cervids (3), a bone regeneration program was co-opted for the annual growth and shedding of antlers. A keratinous sheath covering an osseous core evolved in antilocaprids/pronghorns (4) and bovids (5), although the sheath is annually shed in pronghorns and permanent in bovids. The distant phylogenetic relationship between these families is consistent with an independent origin of the keratinous sheath in the two lineages. The absence of cranial appendages in Moschidae (6) indicates a secondary loss. Phylogeny and timescale follow Hassanin et al. [10].

**Figure 2:**
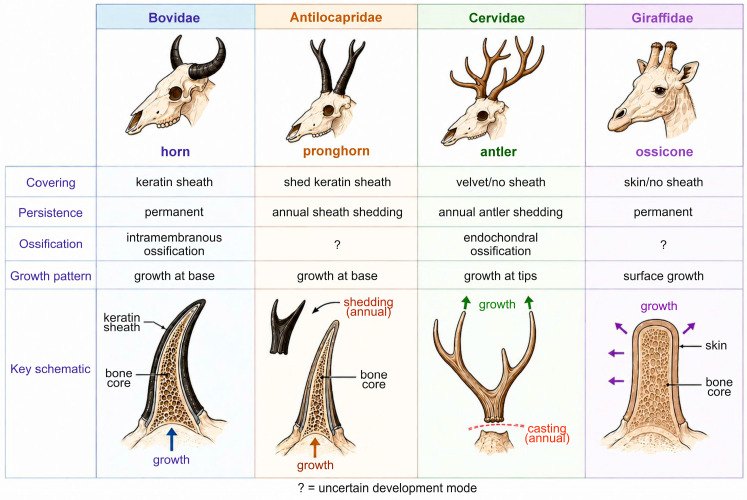
Comparative anatomy and development of the four types of ruminant cranial appendages: bovid horns, pronghorns, cervid antlers, and giraffid ossicones.

Many members of the Ruminantia suborder display headgear structures in the form of osseous cranial appendages, some of which are covered by keratinous sheaths [1,2,3,4]. Additionally, ruminants constitute the only group of contemporary mammals where such paired cranial appendages are found. These osseous and keratinous appendages can be observed among representatives of four out of the six currently existing ruminant families (Figure 1). The osseous cranial appendages in ruminants presumably originated more than 20 million years ago (Mya) and underwent substantial morphological and functional transformations throughout the evolutionary history of this group. Within individual families, the structure of these appendages varies, leading to the identification of four distinct types: permanent horns in the bovid family (Bovidae), pronghorns with deciduous keratin sheath in the American pronghorn (*Antilocapra americana*), antlers in the deer family (Cervidae), and ossicones in giraffids (Giraffidae). A comparative overview of the four types of ruminant cranial appendages is presented in Figure 2. Although colloquially referred to as “horns,” permanent and deciduous horns and giraffid ossicones are designated by distinct technical terms to reflect their differing anatomy and developmental origins. In this review, the term “cranial appendages” is used as a general, inclusive designation for all appendages types of ruminants (horns, antlers, pronghorns, and ossicones). When referring specifically to Bovidae, the term “horn” denotes the entire cranial appendage, comprising both the osseous core and the keratinous sheath.

Cranial appendages serve as ornaments and weapons [1,2]. They are used as weapons in fights between individuals of the same species, particularly among males, and they play a role in establishing social dominance and hierarchy. They may also function as visual signals, allowing individuals to assess an opponent’s strength, age, or physical condition without engaging in direct combat. Horns and antlers can also be used for defense against predators, although predator defense is not generally considered the primary selective force driving their evolution [2]. Sexual selection and intraspecific competition among individuals of the same species are considered the main evolutionary drivers of cranial appendages in ruminants.

The shape of horns and antlers is closely related to the fighting techniques employed by a given species [2]. The long, straight horns of antelopes, especially those of the oryx, function as effective piercing weapons, but they can also be used for hooking and side-slashing attacks. Backward-curved horns facilitate lifting and displacing opponents during combat, whereas the massive horns of rams can be used to destabilize or knock down rivals. Solitary species typically possess sleek and straight horns, highly effective in combat and serving as a means of defense against attackers [3]. Conversely, the horns of social species are typically twisted and adorned with grooves, proving advantageous during battles with other males for hierarchy establishment [3]. Similarly, the antlers of cervids are typically branched, primarily serving the purpose of combat between males. Male cervids possessing reduced, unbranched antlers are capable of delivering powerful and potentially injurious strikes.

The evolutionary origin of ruminant cranial appendages has been debated since the nineteenth century. Two alternative hypotheses have been proposed. The common-origin hypothesis, first proposed by the French anatomist Étienne Geoffroy Saint-Hilaire, suggests that horns, pronghorns, antlers, and ossicones represent evolutionary modifications of a single ancestral cranial appendage rather than independent innovations [5]. In contrast, the independent-origin hypothesis interprets these structures as analogous features that evolved convergently in different pecoran lineages under similar selective forces [2,4].

Throughout most of the twentieth century, the pronounced anatomical and developmental differences among the four appendage types, including differences in ossification, growth pattern, persistence, branching, and keratinization, were considered strong evidence for convergent evolution. Consequently, horns, pronghorns, antlers, and ossicones were generally regarded as analogous rather than homologous structures [2,4]. However, proponents of the common-origin hypothesis argued that ruminant cranial appendages originated only once and were subsequently modified or secondarily lost in different evolutionary lineages [6,7,8]. During the last two decades, advances in phylogenomics, comparative transcriptomics, and single-cell technologies have substantially changed this perspective. The identification of shared developmental pathways, common regulatory genes, and a shared cranial neural crest origin has provided independent molecular evidence supporting a single evolutionary origin of pecoran cranial appendages and subsequent diversification in different evolutionary lineages. Despite the obvious differences among the four cranial appendage types, they also share several morphological characteristics. All are paired, usually symmetrical, osseous outgrowths of the frontal region of the skull, covered by skin during development and largely restricted to ruminants.

Phylogenetic analyses also provide a framework for reconstructing the evolutionary history of cranial appendages (Figure 1). Current phylogenetic evidence indicates that the first osseous cranial appendages evolved near the end of the Oligocene or the beginning of the Miocene, approximately 23.3–20.8 Mya, during the early radiation of Pecora [9]. Because cranial appendages evolved after the divergence of Tragulidae from the remaining ruminants, their absence in tragulids is regarded as the ancestral condition. The earliest cranial appendages may have resembled giraffid ossicones. These ancestral structures were inherited by descendant pecoran lineages and subsequently modified, giving rise to the remarkable diversity of cranial appendages observed today. Phylogenetic analyses further suggest that cranial appendages were secondarily lost only twice during ruminant evolution: once in Moschidae and once in the water deer (*Hydropotes inermis*) within Cervidae.

Reconciling the apparent conflict between morphological and molecular evidence is one of the principal aims of this review. We examine the available anatomical, paleontological, developmental, and molecular evidence to evaluate whether the different forms of ruminant cranial appendages are homologous structures inherited from a common ancestor or analogous structures that evolved independently.

## 2. Horns—Bovidae

**Figure 3:**
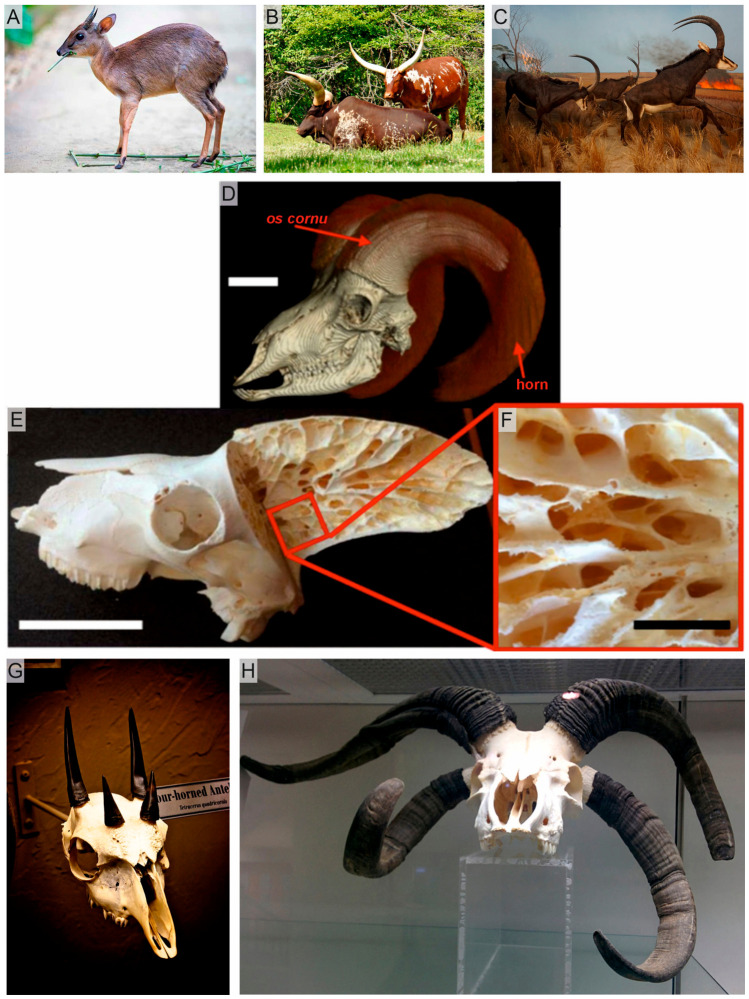
Horns in the family Bovidae. (**A**) The royal antelope (*Neotragus pygmaeus*) has the smallest horns within Bovidae. (**B**) Domestic cattle (*Bos taurus*), represented here by the African Watusi breed, possess some of the longest horns in the family. (**C**) The sable antelope (*Hippotragus niger*) displayed at the Milwaukee Public Museum; this species possesses the longest horns among antelopes. (**D**) Horn structure in the Canadian bighorn sheep (*Ovis canadensis*). A 3D scan illustrating the robust osseous horn core (*os cornu*) that supports the keratinous horn sheath; scale bar = 10 cm. (**E**) Skull of a Canadian bighorn sheep with a sectioned osseous horn core, revealing a pneumatized internal structure containing numerous cavities; scale bar = 10 cm. (**F**) Close-up view of the cavities within the osseous horn core; scale bar = 2.5 cm. (**G**) Skull of the four-horned antelope (*Tetracerus quadricornis*). (**H**) Skull of the four-horned Jacob sheep. The sources of the images are provided in Appendix A.

Typical, permanent horns are found among members of the extensive Bovidae family, which comprises 143 species, including domestic cattle, bison, antelopes, sheep, goats, and ibexes [11]. These animals are also known as bovids or hollow-horned ruminants. The horns are unbranched, typically curved or spirally twisted, and are commonly present in males. In females, horn presence is more variable, ranging from well-developed but usually smaller horns to complete hornlessness in some species. In the greater kudu (*Tragelaphus strepsiceros*), horns are present exclusively in males [12]. The shortest horns, measuring only 3 cm in length, are found in the royal antelope (*Neotragus pygmaeus*) (Figure 3A) [1]. The longest horns, reaching lengths of up to 150 cm, are observed in domestic water buffalo (*Bubalus bubalis*) and African Watussi cattle (*Bos taurus*) (Figure 3B), while among antelopes, the sable antelope (*Hippotragus niger*) possesses horns up to 122 cm long (Figure 3C) [1]. Species inhabiting colder climates tend to possess smaller horns than species occurring in warmer regions. Accordingly, horns have been hypothesized to contribute to thermoregulation through heat dissipation because of their extensive vascularization [13,14]. This hypothesis is supported by observations that horned cows exhibit lower eye temperatures than hornless individuals [15].

Unlike males, whose horns are widely understood as weapons shaped by intrasexual selection for competition over mates and territories [16], the adaptive significance of horns in female bovids has long been debated. Using comparative phylogenetic analyses, it was shown that a composite measure of conspicuousness, calculated from habitat openness and shoulder height, was the strongest predictor of horn presence in females, with female territoriality serving only as a secondary driver in certain small species, such as duikers [17]. This conclusion was further refined by showing that female horn length is influenced by a combination of sociality, territoriality, and male horn size. Nevertheless, natural selection imposed by predation remains the primary evolutionary force driving horn development in female bovids [18].

Horns are not shed during an individual’s lifetime and consist of two main components: the keratinous sheath that encases the osseous horn core (Figure 2). Between the keratinous sheath and the bone, there is a layer of connective tissue that is vascularized and innervated [19]. The keratinous sheath is an epidermal derivative composed of accumulating keratinized, dead cells. The osseous horn core, also known as the *os cornu*, is bone tissue attached to the frontal bones (Figure 3D–F).

**Figure 4:**
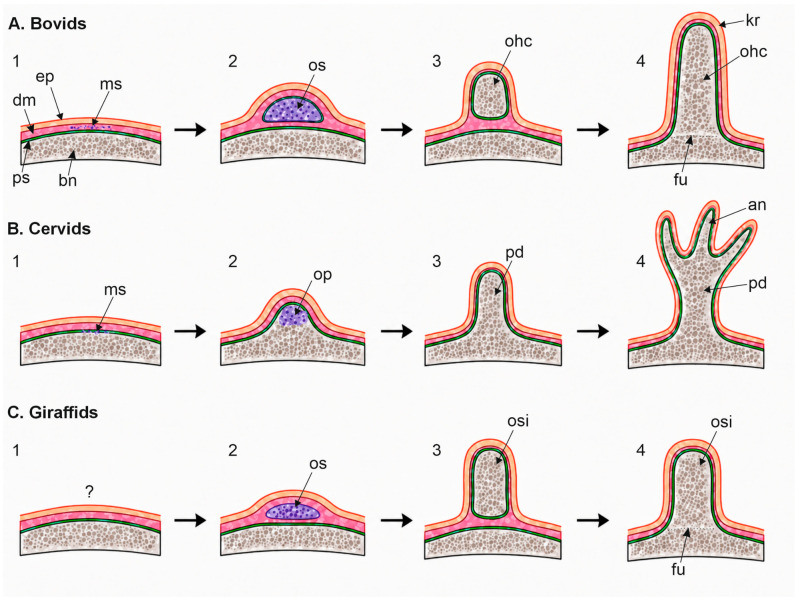
**Schematic comparison of the early development of bovid horns, cervid antlers, and giraffid ossicones.** (**A**) **Bovids.** (1) Mesenchymal progenitor cells (ms) contributing to the future horn core become localized within the dermis (dm), between the epidermis (ep) and the periosteum (ps) of the frontal bone (bn). (2) These cells differentiate into osteoblasts and establish an independent dermal ossification center (os) through intramembranous ossification. (3) Bone deposition gives rise to a young osseous horn core (ohc), which is initially separated from the underlying frontal bone. (4) The horn core subsequently fuses with the frontal bone (fu, fusion site), while the overlying epidermis undergoes intense keratinization and forms the permanent keratinous sheath (kr). (**B**) **Cervids.** (1) Mesenchymal progenitor cells (ms) responsible for pedicle formation are located within the antlerogenic periosteum of the frontal bone. (2) Their activation and osteogenic differentiation initiate formation of the pedicle primordium through the activity of bone-forming cells (op). Unlike the bovid horn core, the pedicle does not form as a separate dermal ossification but develops directly from the frontal bone. (3) The pedicle (pd) elongates as a continuous osseous outgrowth of the frontal bone. (4) The antler (an) subsequently develops from the apex of the permanent pedicle. (**C**) **Giraffids.** (1) The cellular source and earliest stages of ossicone primordium formation remain insufficiently understood. (2) The ossicone primordium develops within the dermis as an ossification center (os) that is initially independent of the underlying skull bones. The precise mode of ossification remains debated, with both intramembranous and cartilage-associated mechanisms having been proposed. (3) The primordium enlarges to form an immature ossicone (osi), which remains separated from the skull during early development. (4) The ossicone subsequently fuses with the underlying frontal and parietal bones (fu, fusion site) and remains permanently covered by skin.

During the fetal period in cows, two thickenings form in the frontal region that do not yet have osseous elements [20]. After birth, beneath the skin in this area, paired accumulations of connective tissue develop, from which small bones emerge, unconnected to the frontal bones (Figure 4A). The osseous horn core develops independently of the frontal bones through direct dermal (intramembranous) ossification, without a preceding cartilaginous template as seen in antlers [13]. At approximately 2 months of age, the osseous cores of the horns fuse with the convex surfaces of the frontal bones [21]. The formed core begins to elongate, and the skin above it begins to keratinize, forming a dead, horny layer that encases the core like a sheath. Already during the fetal period, the skin in the region of future horn development is thicker than in other areas on the head. At around 6 months of age, the horn bones undergo pneumatization, meaning they fill with air from the frontal sinuses [19]. Keratinization on the horn’s surface occurs throughout life, leading to continuous horn growth. Horn growth rates can reach up to 22 cm annually, as demonstrated in Canadian bighorn sheep (*Ovis canadensis*) [22]. The growth of the horn sheath is made possible by the presence of a specialized area at the base where epithelial cells proliferate and undergo intense keratinization [23]. Thus, horns are characterized by basal growth (Figure 2). The keratinized sheath at the widest, proximal part of the horn is the youngest, whereas the distal part of the horn is the oldest. The spiral growth of horns results from differences in the rate of keratin layer deposition on both sides of the horn’s base. The rate of cell division and keratinization varies over time and depends on factors such as the season, health status, or pregnancy [23]. Consequently, transverse growth lines are visible on the horn’s surface. The horn sheath is never shed, but its outer layers may slough off. The removal of horn osseous horn cores rudiments in bovids does not halt the tendency of the skin to form horn structures [23]. In this area, the skin ceases to produce hair, begins to synthesize keratin, and leads to the formation of a new osseous knob. Conversely, removing the skin from the horn development area completely blocks the horn formation process. Therefore, the skin plays a central role in controlling horn growth.

Horns are of considerable importance in cattle, sheep, and goat husbandry. They serve as secure attachment points for harnesses in draft cattle, provide defense against predators while grazing, and aid in temperature regulation in warm climates.

Horned animals may pose a risk to conspecifics and handlers, require more space during transport, and necessitate specialized equipment, such as adapted feeders and waterers. For this reason, some breeders use dehorning, which improves safety during handling and transport and reduces injury risk [24]. Dehorning may be performed mechanically, thermally using heated irons, or chemically with caustic agents, preferably during the first weeks of life, before the osseous horn cores fuse with the frontal bones. Hornless cattle can also be obtained through crossbreeding. There are cattle breeds, such as Aberdeen Angus and Brangus, that are naturally hornless due to the presence of a dominant *P* allele. Individuals are hornless when homozygous *PP* or heterozygous *Pp*, while homozygous pp individuals have horns [25]. Advanced genetic engineering methods are also being developed to produce hornless animals, such as cattle [26]. Among cattle, a mutation is also known that leads to the formation of underdeveloped, non-fused horns called scurs.

While most bovids have a single pair of horns, there are instances of animals having two pairs of horns. The Asian four-horned antelope (*Tetracerus quadricornis*) is a species naturally possessing two pairs of horns (Figure 3G). Furthermore, the occurrence of multiple horns (polycerate) is also known among domestic cattle, sheep, and goats [27]. Examples include the four-horned goat (Vierhornziege), the multi-horned Jacob sheep, the Manx Loaghtan sheep (Figure 3H). There have even been documented cases of goats with up to eight horns. It was shown that multiple horns are associated with defective *HOXD1* function. Haploinsufficiency of this gene results in the splitting of horn bud primordia [27].

## 3. Pronghorns—Antilocapridae

**Figure 5:**
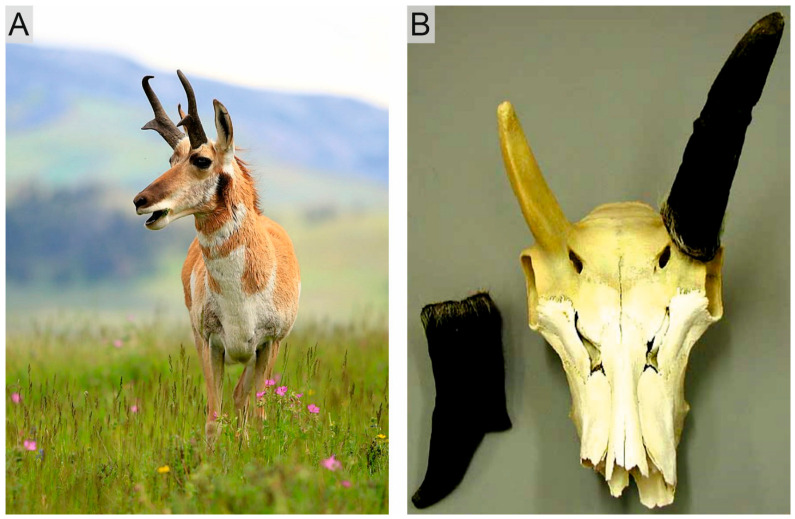
Pronghorns (*Antilocapridae*). (**A**) An adult male American pronghorn (*Antilocapra americana*) in Yellowstone National Park. The characteristic anterior prong of the horn is clearly visible. (**B**) Skull of the American pronghorn. On the left side, the keratinous horn sheath has been removed, exposing the underlying osseous core. The sources of the images are provided in Appendix A.

The pronghorn (*A. americana*) is the sole living representative of the family Antilocapridae (Figure 1 and Figure 5A) [28]. This species is primarily found in the United States, but it also inhabits limited areas in Mexico and Canada. The distinct pronged horns of pronghorns serve both communicative functions within the herd and are used in male-male combat and defense [29]. Their horns possess several unique characteristics, including lateral flattening, annual shedding and regrowth of the keratinous sheath, and a bifurcated keratinous sheath overlying an unbranched osseous pronghorn core. Due to these unique features and the presence of a front prong, in English, pronghorns’ horns are referred to as “pronghorns” to distinguish them from the typical horns of bovids [2]. Pronghorn horns are present in males and only in about 30% of females, and in females, they are notably smaller [13]. The horns of males reach their largest size during the rutting season, which occurs in September. At this time, the androgen levels in the blood of males are at their highest. Shortly after the rutting season, in autumn or early winter, the keratinous sheaths are shed, leaving the osseous cores covered with hair-covered skin. During this period, the skin keratinizes, and the process of regrowing the keratinous sheath continues until summer [13].

Little is known about the development of bones inside the pronghorns [13]. The overall organization and growth pattern are shown in Figure 2. It is unclear whether the osseous core forms as a separate ossification of the skin, similar to bovids, or as an osseous extension of the frontal bones, as in cervids. Pronghorns develop in the first months of life, and their keratinous sheaths are shed during the first winter. Between the osseous core and the keratinous sheath lies the skin with connective tissue and epidermis, which keratinizes, forming the sheath. Hairs grow on the skin’s surface beneath the sheath, causing the sheath to separate and shed. Subsequently, the epidermis undergoes intense keratinization, forming a new sheath that incorporates the hair previously growing beneath the old sheath. The osseous core remains unbranched, while within the pronghorn, there are two keratinization centers—one at the tip and one on the anterior surface. As a result, the keratinous sheath takes on a forked shape [13].

## 4. Antlers—Cervidae

**Figure 6:**
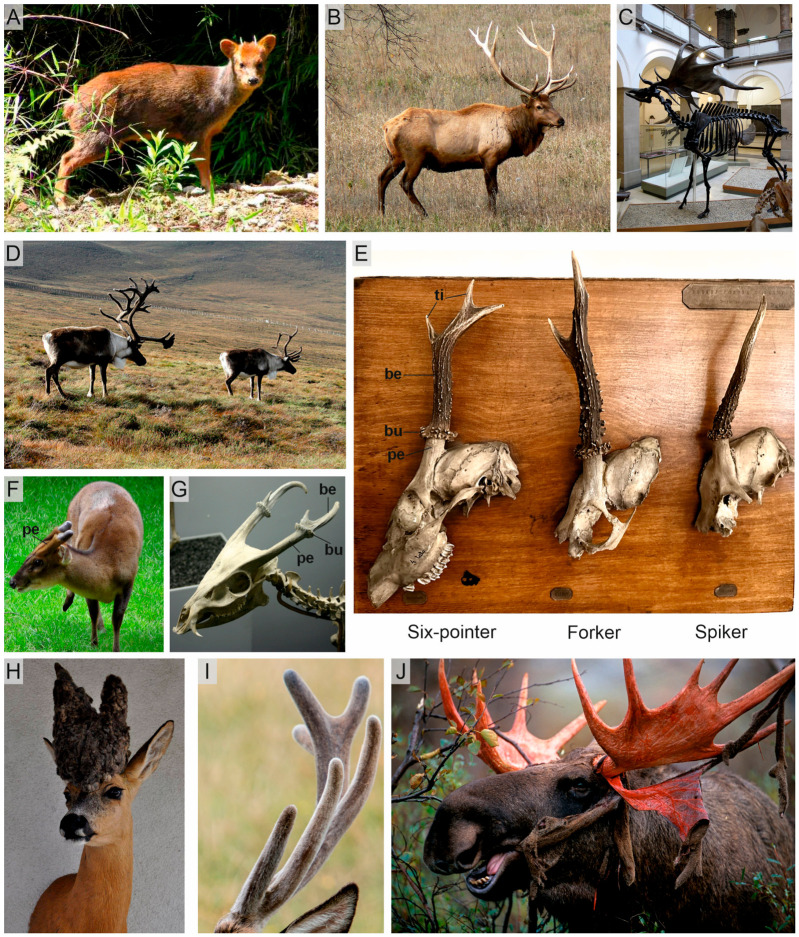
Antlers in the family Cervidae. (**A**) The southern pudu (*Pudu puda*), which possesses the smallest antlers among extant cervids. (**B**) The Canadian elk or wapiti (*Cervus canadensis*), which develops the largest antlers among extant cervid species. (**C**) A skeleton of the giant Irish elk (*Megaloceros giganteus*) displayed at the Palaeontological Museum in Munich; this extinct species possessed the largest antlers known in the evolutionary history of Cervidae. (**D**) A male (left) and female (right) reindeer (*Rangifer tarandus*), the only extant cervid species in which females regularly develop antlers. (**E**) Historical specimen from the Collection of the Department of Comparative Anatomy, Jagiellonian University, illustrating the morphology of the European roe deer (*Capreolus capreolus*) antlers. From left to right: a six-pointer, forked antlers, and spike antlers. The following structures are indicated: pe, pedicle; bu, burr; be, main beam; ti, tine. (**F**) The Reeves’s muntjac (*Muntiacus reevesi*). (**G**) Skull of the Reeves’s muntjac showing the elongated pedicle (pe) and short antler beam (be). (**H**) A velvet buck (European roe deer) exhibiting malformed antlers. (**I**) A deer antler covered in velvet. (**J**) A Eurasian moose (*Alces alces*) consuming shed velvet. The inner surface of the velvet reveals a dense network of blood vessels that are lost when the velvet is shed. The sources of the images are provided in Appendix A.

Antlers are composed of exposed, branching, dying bone when mature, and are unique among ruminant appendages in their ability to undergo complete periodic regeneration [30]. They are found in the deer family (Cervidae), which encompasses 55 species [11]. Antler size varies considerably among cervid species. Among extant cervids, the smallest antlers (2.5–3.5 cm) occur in the Asian tufted deer (*Elaphodus cephalophus*) and in the South American pudus (*Pudu* spp.), whose antlers range from 6 to 9 cm in length [31,32]. The largest antlers belong to the Canadian elk (*Cervus canadensis*), reaching lengths of about 150 cm and weighing up to 18 kg (Figure 6A,B) [33]. In the past, the largest antlers were found in the giant deer also called Irish elk (*Megaloceros giganteus*), which inhabited Eurasia during the Pleistocene epoch (Figure 6C). Their span could reach up to 3.6 m, with a weight of 40 kg [34]. The Irish elk possessed exceptionally large antlers; however, these structures were not disproportionately large relative to body size, but instead fell within the allometric trends observed in large deer.

**Figure 7:**
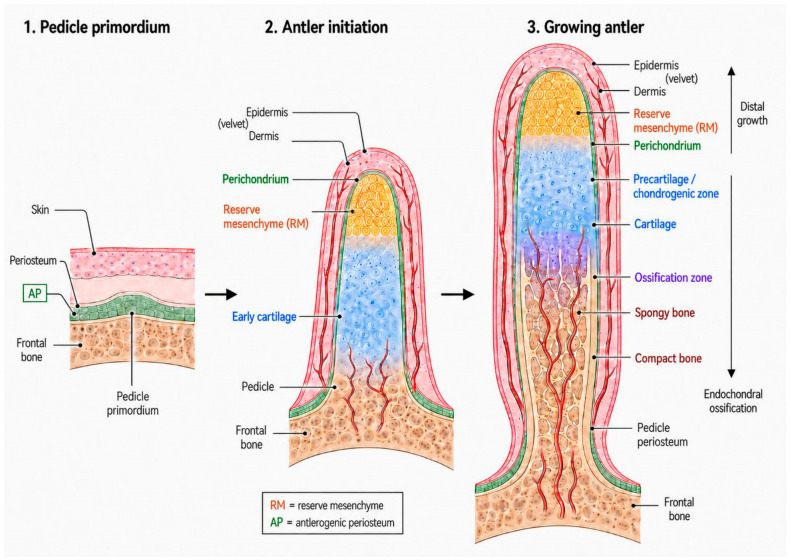
Schematic overview of antlerogenesis and longitudinal organization of the growing deer antler. (**1**) Formation of the pedicle primordium from the frontal bone beneath the antlerogenic periosteum (AP). (**2**) Initiation of antler growth following differentiation of the periosteum into perichondrium and establishment of the reserve mesenchyme and cartilaginous primordium. (**3**) Longitudinal organization of the growing antler showing the reserve mesenchyme, precartilage/chondrogenic zone, cartilage, ossification zone, spongy bone, compact bone, and pedicle periosteum. Antler elongation occurs by distal growth, whereas endochondral ossification progressively replaces cartilage with bone.

In most species, antlers are exclusively found in males, and their development is dependent on the level of androgens in the bloodstream. Reduced androgen levels following prepubertal castration may prevent antler formation, whereas exogenous testosterone may promote antler growth in females [35]. Androgens activate a population of mesenchymal progenitor cells located in the antlerogenic periosteum (Figure 7) [36]. These cells are present in both sexes but become activated only under sufficient androgen stimulation, explaining why antlers normally develop only in males. The reindeer/caribou (*Rangifer tarandus*) are the only species where antlers are present in both males and females (Figure 6D) [37]. Nevertheless, they attain larger sizes in males. Castration or the administration of androgen-inhibiting substances does not inhibit antler development in reindeer [38]. In other cervids (such as deer, elk, and moose), the androgen receptor (AR) activates cyclin D1 expression (the *CCND1* gene is a key cell-cycle regulator leading to cell proliferation) only at high testosterone concentrations, which are reached exclusively in males. In reindeer, however, a specific mutation in the regulatory region of the *CCND1* gene created a novel androgen receptor binding site that becomes active even at the low androgen levels typical of females, thereby enabling antler growth regardless of sex [39]. Reindeer inhabit areas with sparse vegetation, often covered by snow for most of the year. In female reindeer, antlers are used during foraging to scrape away snow and access lichens and other low-growing plants. In winter, after most males have cast their antlers, female reindeer typically retain theirs and use them in competition for limited food resources. This period coincides with pregnancy in females, during which elevated circulating estrogen levels are thought to inhibit antler casting [40].

Antlers are attached to osseous pedicles which are outgrowths of the frontal bones [23,41]. The pedicles are covered in skin of the antler base. The main part of the antler is the long shaft known as the beam, which has branches called tines (Figure 6E). At the base of the antlers, there is a thickening referred to as the burr, rosette or coronet. A unique shape of antlers is found in muntjacs (*Muntiacus*), where the hairy base containing the pedicles is elongated, and the beam on it is relatively small (Figure 6F,G).

Beyond simple variation in tine number, the branching pattern of antlers itself may contain important phylogenetic information. In a recent comparative study, antler grooves, which reflect growth direction and vascular impressions, were used to project the branching directions of tines onto the burr and to identify homologous antler elements across cervids [42]. Ancestral-state reconstruction suggested that the most recent common ancestor of extant deer possessed two-pointed antlers composed of a brow tine and a lower beam, whereas three-pointed antlers evolved independently in Cervini and Capreolinae subfamilies. Thus, similar external antler forms may be homoplastic, and detailed comparison of tine homology can help link antler morphology with molecular phylogeny and the classification of fossil cervids.

Antler size increases allometrically with body mass [43]. The first set of antlers in a young deer is typically unbranched, forming what is called spikes, and a male with such antlers is called a spiker [23,41]. These antlers are subsequently cast, and in the following year a new set develops, usually with branches. A male deer with two tines on each beam is called a forker (Figure 6E). A six-pointer has three apexes (tips) on each beam. Males with an increasing number of tines are called eight-pointers, ten-pointers, twelve-pointers, and so on. Typically, the number of tines increases with age, reaches its peak at around 10–14 years. In the past, it was believed that the number of tines on antlers indicated the age of the male. However, it is now known that the development of antlers reflects the nutritional status and overall health of the animal [44]. In hunting jargon, the antlers of moose are called paddles due to their flattened shape, whereas the antlers of deer are referred to as crowns. In males with hormonal disorders, particularly those associated with testicular dysfunction, cryptorchidism, or castration before completion of antler development, antlers may fail to undergo the normal cycle of growth and casting. Instead, they remain permanently covered with velvet, continue growing in an irregular and uncontrolled manner, and form deformed, tumor-like masses known as perruque antlers (Figure 6H) [45].

The development of antlers, known as antlerogenesis, begins during the fetal period in males (Figure 7) [46]. The pedicle primordia take the form of spongy bone located beneath the periosteum of the frontal bone. This indicates that, unlike in Bovidae, the pedicles are extensions of the frontal bone itself and not separate ossifications (Figure 4B). Formation of the cartilaginous tissue is involved in antler development, in contrast to horns in Bovidae, indicating significant developmental differences between these structures. Transplanting primordia of pedicle, called antlerogenic periosteum (AP), to another location results in the growth of antlers in that new location [47]. Complete removal of the primordia prevents antler development altogether [23]. In males, the pedicle primordia contain numerous osteoblasts, which are absent in females. The antlerogenic periosteum of females remains quiescent because of the lack of the hormonal requirements necessary for pedicle formation. After reaching sexual maturity, pedicle growth is arrested, and the periosteum is transformed into a perichondrium, which induces the formation of cartilage tissue that subsequently develops into antlers (Figure 7) [41,48]. During their development, antlers are highly vascularized, possess significant sensory innervation, and are the fastest-growing tissue in animals, with growth rates exceeding 2 cm per day [41,49]. This rapid growth of antlers is due to the proliferation of cartilage cells. Growth tips (apexes) are located at the ends of the antlers. The rapid growth of cartilage in antlers is sustained by a robust blood supply. This is a unique situation, as cartilage is usually not vascularized in other parts of the body. In antler development, cartilage cells are removed by chondroclasts and replaced by osteoblasts, leading to the formation of bone tissue. This tissue elongates, forming the growing antler, with the cartilage being replaced by lamellar bone in a process known as endochondral ossification (Figure 7) [41,48,49]. These developmental characteristics are compared with other ruminant cranial appendages in Figure 2 and Figure 4. In the center of the antler, the bone has a spongy structure, while the peripheral layers of the antler consist of hard bone tissue with an organized structure in the form of cylindrical osteons. The presence of a spongy core is not a universal feature of cervid antlers. In some tropical species, whose antlers may grow aseasonally and be retained for extended periods, the antlers are more compact and may lack a distinct spongy core. The rapid growth of antlers requires large amounts of calcium, which are drawn from the remaining skeletal bones, potentially leading to temporary osteoporosis [41].

Pedicles remain permanently covered in skin, whereas antlers are covered in velvet only during their growth phase [50]. After the antlers have finished growing, the skin covering them, called velvet, dies, and it is shed when the antlers rub against trees and bushes (Figure 6I,J). Along with the shedding of velvet, the blood vessels that nourish the antler bone are also lost. This results in the death of the bone tissue of the antlers [51]. During the period when the antlers are at their peak development and the velvet is shed, the level of androgens in the blood is at its highest. This marks the beginning of the rutting season and mating battles. After the rutting season, testosterone levels decrease, and osteoclasts, which are bone-resorbing cells, begin to form at the base of the antlers on the pedicles [49]. These cells break down the bone tissue, leading to the shedding of the antlers.

Antlers undergo a cyclic process of growth, mineralization, velvet shedding, and casting that is regulated primarily by seasonal fluctuations in androgen levels [49,52,53]. In temperate cervids, this cycle is tightly synchronized by photoperiod [23,52]. Increasing day length stimulates antler growth, whereas peak testosterone concentrations induce antler mineralization and velvet shedding before the rut. Following the breeding season, declining androgen levels trigger osteoclastic resorption at the pedicle–antler junction, resulting in antler casting. In red deer (*Cervus elaphus*), antler growth begins in spring, and velvet is typically shed in July and August when the antlers reach their full size. The rut usually starts in the second half of September, and stags cast their antlers between late February and April. Roe deer (*Capreolus capreolus*) shed their antlers in late October and November, moose (*Alces alces*) cast their antlers in December and January, whereas European fallow deer (*Dama dama*) typically shed their antlers from April to May. South American cervids (e.g., *Pudu* spp.) transferred to European zoos initially retained their Southern Hemisphere antler periodicity until cast, after which the animals gradually synchronized their antler cycle with the photoperiodic regime of the Northern Hemisphere [54].

In many tropical and subtropical species, where annual variation in day length is minimal, antler cycles are less synchronized at the population level [55,56]. Individuals from the same population may simultaneously exhibit growing antlers covered with velvet, fully mineralized antlers, or recently cast antlers. In these species, antler development may occur aseasonally and appears to be influenced more strongly by local environmental factors, such as rainfall patterns, food availability, and individual physiological condition than by photoperiod alone [57,58]. Consequently, antlers may be retained for longer periods, and the timing of casting and regrowth can vary substantially among individuals. For example, in tropical populations of chital (*Axis axis*) and sambar deer (*Rusa unicolor*), all stages of the antler cycle may be observed throughout the year [56,58]. By contrast, temperate cervids exhibit highly synchronized annual antler cycles.

## 5. Ossicones—*Giraffe* and *Okapi*

**Figure 8:**
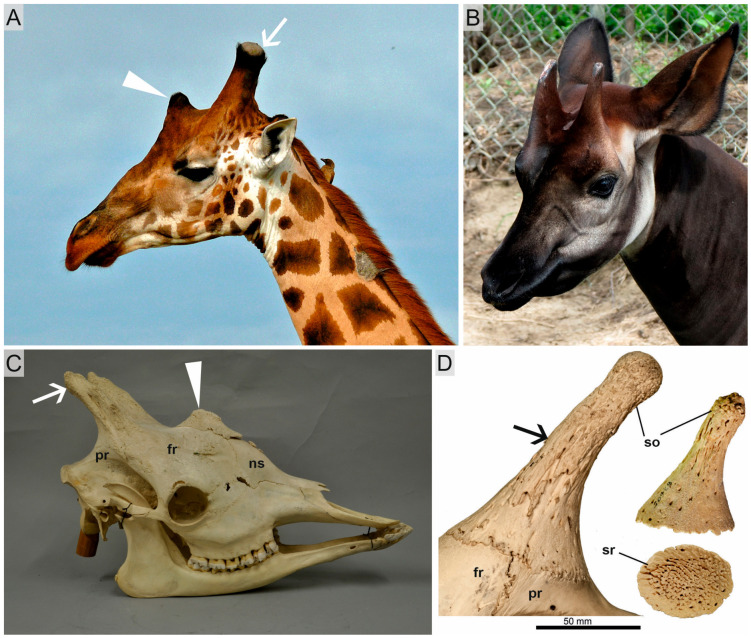
Ossicones in giraffids (Giraffidae). (**A**) Head of the northern giraffe (*Giraffa camelopardalis*) showing a pair of primary ossicones (arrow) and a median ossicone (arrowhead). (**B**) Head of the okapi (*Okapia johnstoni*) displaying a pair of ossicones. (**C**) Giraffe skull at Museum Wiesbaden showing a pair of primary ossicones (arrow) positioned over the frontal (fr) and parietal (pr) bones, as well as a median ossicone (arrowhead) located over the nasal (ns) and frontal (fr) bones. (**D**) Structure of a giraffe ossicone. The ossicone (arrow) arises from the frontal (fr) and parietal (pr) bones. Secondary ossification deposits (so) are visible at its apex. The contact surface (sr) between the ossicone and the underlying skull bones is also visible. The sources of the images are provided in Appendix A.

Traditionally, the family Giraffidae has been considered to comprise two extant genera, *Giraffa* and *Okapia*. The genus *Giraffa* was historically regarded as containing a single species, *Giraffa camelopardalis*, whereas *Okapia* is represented by a single species, *Okapia johnstoni* (Figure 8A,B). However, recent studies support the recognition of four extant giraffe species: *Giraffa camelopardalis* (northern or three-horned giraffe), *Giraffa giraffa* (southern or two-horned giraffe), *Giraffa reticulata* (reticulated giraffe), and *Giraffa tippelskirchi* (Masai giraffe) [59]. All members of this family inhabit Africa and possess osseous appendages, called ossicones, on their skulls, which have the simplest structure among all ruminants [13,60]. The main pair of ossicones, often referred to as the frontal ossicones, is located at the border of the frontal and parietal bones and is present in both sexes but reaches larger sizes in males, measuring up to 18 cm in length. These ossicones thicken due to additional surface growth of bone tissue, secondary ossification, during adulthood in males (Figure 2, Figure 4C and Figure 8C,D) [60]. In addition to the frontal ossicones, additional smaller ossicones may occur in the areas of the naso-frontal, temporal, occipital, zygomatic, and supraorbital regions [13]. Accessory ossicones, especially a single central appendage on the nasal and frontal bones, are common in males but rarely found in females, and their degree of development varies. The ossicones of males serve a purpose during combat, in which males strike each other with their heads. Some suggest that the highly vascularized ossicone bones may have a thermoregulatory role [60].

In giraffes, during prenatal development, rudimentary ossicone primordia surrounded by connective tissue capsules form at the border between the frontal and parietal bones (Figure 4C). A well-developed network of blood vessels separates these primordia from the skull bones. The ossicone primordia arise in the dermal integument independently of the cranial bones, followed by subsequent fusion with the underlying frontal and parietal bones of the skull, which is similar to the cores of bovid horns [61]. The osseous ossicones fuse with the skull bones in males during their fourth year of life and in females during their seventh year [60]. According to some authors, the ossicone primordia are initially cartilaginous and later undergo endochondral ossification [60,61]. However, others state that ossicones arise from the integument’s fibrous connective tissue via fibrocellular/intramembranous ossification in fetal and neonatal giraffes, with occasional foci of transition through fibrocartilage; this is not classic endochondral ossification [62].

In the case of okapi, ossicones are only present in males and also serve for combat and defense (Figure 8B). Similar to giraffes, they develop as cutaneous ossifications and later fuse with the skull bones [61]. In okapi, ossicones fuse with the frontal bones. The distal ends of adult male okapi ossicones protrude above the skin and are not covered by it [63], whereas in giraffes, they remain covered by skin throughout. It has been suggested that the boundary between the region of ossicones covered by skin and the region without skin is significant for considerations regarding the evolution of cranial appendages in ruminants and may correspond to the attachment site of antlers on pedicles [4].

## 6. Paleontological Evidence for the Evolution of Cranial Appendages in Pecoran Ruminants

**Figure 9:**
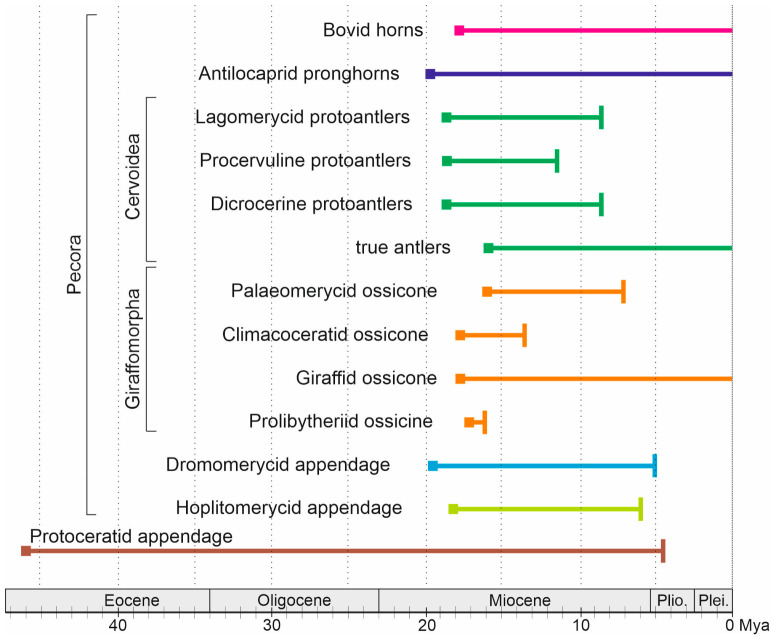
Temporal distribution of the major types of cranial appendages in fossil and extant ruminants and closely related Protoceratids. Horizontal bars indicate the known stratigraphic ranges of representative cranial appendage types based on the fossil record.

The fossil record provides important, although still incomplete, framework for interpreting the evolution of cranial appendages in ruminants. The origin of Ruminantia and the first appearance of cranial appendages were not synchronous events. The earliest ruminants are thought to have appeared approximately 50–55 Mya, during the early to middle Eocene, in North America and Eurasia [64,65]. By contrast, the major radiation of Pecora, the clade that includes Bovidae, Cervidae, Moschidae, Giraffidae, and Antilocapridae, began later, around 30 Mya, during the Oligocene [66]. Molecular divergence probably predates the oldest fossil record by several million years. Cranial appendages are not documented in the earliest ruminants. Instead, they appear relatively abruptly in the fossil record during the early Miocene, approximately 19.5–17 Mya, when different types of headgear suddenly emerged in several pecoran lineages [67]. The temporal ranges of the major cranial appendage types in extant and extinct ruminant lineages are summarized in Figure 9.

This early Miocene interval is therefore critical for understanding the evolution of horns, pronghorns, antlers, and ossicones. The near-simultaneous appearance of diverse cranial appendages in different ruminant lineages has often been linked to ecological and climatic changes. It has been suggested that their evolution may have been promoted by climatic cooling, increasing environmental seasonality, and the expansion of more open habitats, which may have intensified social competition and sexual selection among early pecorans [66,67]. However, this process should not be interpreted as a simple, continuous trend. It was postulated that the evolution and diversification of cranial appendages occurred in distinct evolutionary pulses and was partially interrupted by climatic instability during the early–middle Miocene transition [30]. Thus, the early history of ruminant cranial appendages was probably shaped by a combination of ecological opportunity, sexual selection, phylogenetic constraint, and repeated experimentation in different lineages.

The diversity of extinct pecoran cranial appendages was much greater than that observed among living ruminants. Fossil forms possessed appendages that could be single or paired, unbranched or branched, flattened, twisted, posteriorly inclined, laterally expanded, or arranged in unusual numbers. Some extinct taxa had one, two, or even three pairs of appendages, whereas others developed unpaired, anterior or posterior cranial structures. This diversity indicates that the four major extant categories, bovid horns, antilocaprid pronghorns, cervid antlers, and giraffid ossicones, represent only a restricted subset of a much broader Miocene morphological radiation. Consequently, fossil data do not support a simple linear scenario in which one type of cranial appendage gave rise directly to another. Rather, they point to a complex pattern of parallel, divergent, and possibly convergent evolution.

An additional hallmark of extinct ruminants is the remarkable positional diversity of cranial appendages. Fossil taxa developed headgear on the frontal, supraorbital, parietal, occipital, and nasal regions of the skull, with appendages occurring as paired, multiple paired, or median structures. Such diversity has no counterpart among extant ruminants and emphasizes that the early evolution of cranial appendages involved extensive morphological experimentation across different pecoran lineages.

**Figure 10:**
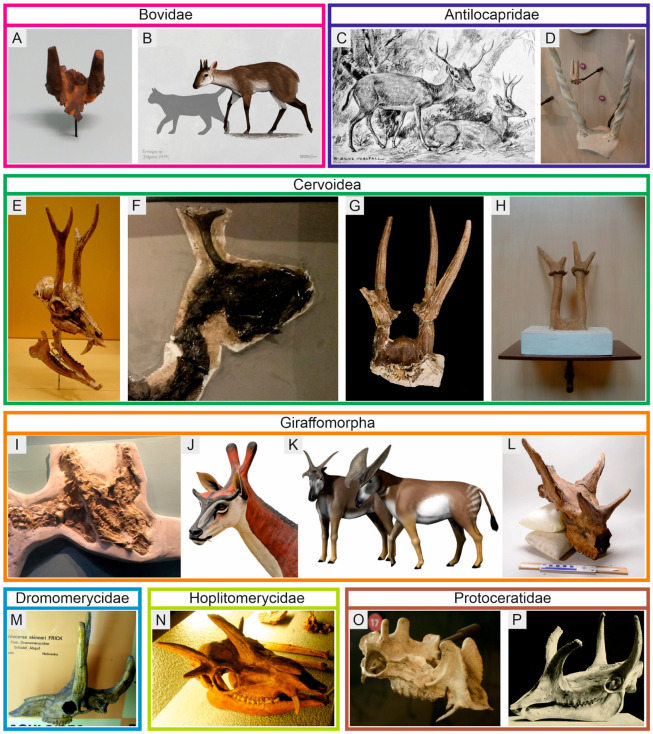
**Fossils and life reconstructions illustrating the diversity and evolution of cranial appendages in extinct ruminants and related artiodactyls.** (**A**) Fossil horn cores (osseous appendage) and skull fragment of *Eotragus sansaniensis*, one of the earliest known bovids. (**B**) Life reconstruction of *Eotragus* compared with a domestic cat for size reference. (**C**) Life reconstruction of the early pronghorns *Ramoceros osborni* (left) and *Cosoryx furcatus* (right). (**D**) Fossil horn core of *Ilingoceros alexandrae*. (**E**) Fossil skull of *Procervulus* (Cervidae) bearing forked protoantlers lacking a burr. (**F**) Fossil skull of *Lagomeryx* (Lagomerycidae) with protoantlers. (**G**) Fossil frontal bones bearing antlers of *Dicrocerus elegans* (Cervidae). (**H**) Fossil antlers of *Euprox furcatus* (Cervidae). (**I**) Fossil skeleton of *Palaeomeryx* (Palaeomerycidae). (**J**) Life reconstruction of *Ampelomeryx ginsburgi* (Palaeomerycidae). (**K**) Life reconstruction of a female (left) and male (right) *Prolibytherium magnieri* (Climacoceratidae) by the author. (**L**) Fossil skull with ossicones of *Giraffokeryx punjabiensis* (Giraffidae). (**M**) Fossil skull cast of *Cranioceras skinneri* (*Procranioceras skinneri*). (**N**) Fossil skull of *Hoplitomeryx matthei*. (**O**) Skull of *Protoceras celer*. (**P**) Fossil skull of *Syndyoceras*. The sources of the images are provided in Appendix A.

Bovidae are commonly defined by the presence of permanent, unbranched osseous horn cores covered by a persistent keratinous sheath, although some early hornless ruminants with bovid-like dental characters have also been discussed as possible stem bovids or very primitive bovids. One example is *Namibiomeryx*, which has been interpreted as a stem bovid or a primitive hornless bovid [68]. The earliest well-documented horned bovids is *Eotragus* (Figure 10A,B). This species is known from early Miocene sites in France and Pakistan, approximately 18–17 Mya, and *Namacerus* from Namibia, approximately 17.5 Mya [69,70]. These early bovids possessed small, conical osseous horn cores. The transition from hornless to horned bovids remains poorly documented, but the available fossil evidence suggests that the bovid lineage had already diverged from other pecoran branches before the appearance of true horns. This supports the interpretation that bovid horns evolved within the bovid lineage and were not inherited as fully formed structures from a common headgear-bearing ancestor shared with cervids, giraffids, or antilocaprids.

Moschidae are particularly important in this context because living musk deer lack horns, antlers, pronghorns, and ossicones. Fossil moschids, including *Micromeryx* and *Hispanomeryx*, known from the middle to late Miocene, also lack cranial appendages [71,72]. These taxa indicate that the moschid lineage had already adopted a hornless condition during the Miocene. However, the fossil record does not currently document moschid ancestors with cranial appendages. Therefore, the hypothesis that Moschidae secondarily lost headgear is not directly demonstrated by paleontological evidence. Instead, it is mainly inferred from phylogenetic and developmental considerations, especially from the close relationship of Moschidae to Bovidae and from the broader distribution of cranial appendages among Pecora. At present, paleontology alone cannot determine whether moschids retained an ancestrally hornless condition or lost an earlier appendage-forming developmental potential.

Antilocapridae evolved in North America, where they underwent a substantial Miocene and Pliocene radiation. In ecological terms, they occupied roles broadly comparable to those of Old World bovids. Their extinct representatives show remarkable diversity in cranial appendage morphology. Some taxa possessed relatively simple horns, whereas others developed highly unusual headgear, including four or even six horns. In several forms, the appendages had elongated, forked, flattened, palmate, or antler-like shapes. For example, *Ramoceros* bore long and strongly branched appendages resembling antlers in general outline, whereas *Ilingoceros* had straight, spiraled horns ending in forked tips (Figure 10C,D). *Merriamoceros* possessed flattened, fan-shaped appendages, and *Hayoceros* had four horns [73,74]. *Proantilocapra*, from the Miocene of North America, already showed a morphology broadly comparable to that of the living pronghorn, *A. americana* [75]. This diversity indicates that antilocaprid pronghorns were not merely simplified versions of bovid horns, but part of an independent North American radiation of cranial appendages. Because keratinized sheaths are not preserved in the fossil record, it is difficult to determine whether fossil osseous horn cores were covered by a keratinous sheath.

The early evolution of cervid antlers is complex and has undergone substantial reinterpretation in recent years. The earliest antler-like cranial appendages, commonly referred to as protoantlers, are known from early Miocene cervoids such as *Procervulus*, one of the most basal members of Cervidae and among the earliest ruminants bearing antler-like structures (Figure 10E) [76]. The protoantlers of *Procervulus* were forked but differed markedly from the true antlers of extant deer. They lacked a burr (coronet), the osseous ring at the antler base that characterizes modern cervids, and were probably permanently covered by skin rather than undergoing velvet shedding [77].

Protoantlers were long considered to be permanent (non-deciduous) cranial appendages. However, subsequent anatomical and histological studies have demonstrated that even the earliest fossil cervids exhibited a cyclical process of appendage shedding and regeneration, indicating that the antler cycle originated much earlier than previously assumed [78]. This interpretation is supported by several histological and anatomical features preserved in fossil protoantlers, including osteoclastic resorption at the antler base, marking the future site of abscission; extensive secondary bone remodeling characteristic of modern antlers; vascularization patterns and osteonal organization comparable to those of extant cervid antlers; histological evidence of regeneration following previous shedding events; and the presence of well-defined abscission scars on shed protoantlers [78].

Early protoantler-bearing cervoids included procervulines and lagomerycids, such as *Procervulus*, *Ligeromeryx*, and *Lagomeryx*, which inhabited Europe during the early Miocene, approximately 18–16 Mya (Figure 10F). Histological evidence indicates that all of these taxa underwent repeated cycles of protoantler shedding and regeneration, despite lacking the fully developed burr that characterizes the antlers of extant deer [78].

A key question is whether all protoantlers were homologous with true cervid antlers or whether similar appendages evolved more than once among early cervoids. Some early Miocene forms show evidence of spontaneous and complete shedding, as indicated by regular, porous, and rugose abscission surfaces at the proximal end of the appendage. This suggests that the mechanism of appendage shedding had already appeared in some early or middle Miocene cervoids [77]. However, the earliest protoantlers were less strongly mineralized and may have remained vascularized and living for longer periods. Anatomical and histological differences among protoantler-like structures have led to the suggestion that at least some of them may not have been strictly homologous, but may instead represent independently evolved cranial appendages within the broader cervoid radiation [30].

More advanced protoantlers are known from dicrocerines such as *Dicrocerus* (Figure 10G). In these forms, a partially developed coronet-like structure was present, and the appendage cycle appears to have approached the condition seen in true antlers. The cycle probably included a phase of shedding of velvet-like skin, followed by a phase in which the hard, bare, dead protoantler was retained before being cast [79]. True antlers, defined by the presence of a well-developed coronet, are recorded in *Euprox* from middle Miocene sites of Eurasia, approximately 15 Mya [30]. *Euprox* is therefore important as one of the oldest true cervids with antlers approaching the modern cervid condition (Figure 10H). The fossil record of cervids thus suggests a gradual acquisition of key antler features, branching, periodic shedding, velvet loss, death of the mineralized appendage, and a well-developed coronet, but this acquisition may have occurred through a mosaic rather than a strictly linear process.

Giraffomorpha provide another important example of Miocene cranial appendage diversity. This clade includes Palaeomerycoidea, represented by Palaeomerycidae, and Giraffoidea, including Giraffidae, Prolibytheriidae, and Climacoceratidae. Giraffomorphs are defined not only by the presence of ossicone-like appendages but also by additional cranial and postcranial skeletal characters [80]. Palaeomerycidae, which lived approximately 16–7 Mya, included genera such as *Palaeomeryx*, *Ampelomeryx* and *Xenokeryx* (Figure 10I,J). These animals possessed paired frontal or supraorbital ossicone-like appendages, whose homology with true giraffid ossicones remains debated, and, in some cases, a single branched occipital appendage involving elongation and modification of the nuchal plane and supraoccipital region.

Prolibytheriidae were characterized by massive aliform, or wing-like, frontal ossicone-like appendages that were strongly flattened and extended laterally over the braincase and beyond the nuchal crest. These forms lived approximately 17–16 Mya and represent one of the most distinctive experiments in ruminant cranial appendage evolution [81]. Climacoceratidae also possessed large ossicone-like appendages, some of which resembled antlers or thorn-covered plant stems, whereas others formed crescent-like structures (Figure 10K) [82]. Early giraffids such as *Canthumeryx* and *Giraffokeryx*, dated to approximately 18–16 Mya, showed a more primitive condition (Figure 10L). In this genus, the frontal bone bore a pair of small, laterally expanded protuberances resembling primitive ossicones: flat, triangular-based structures positioned above the orbits and oriented laterally, rather than upward or backward as in more derived giraffids [83]. These fossils indicate that ossicones, similarly to horns and antlers, passed through a series of early morphologies before reaching the condition seen in living giraffes and okapis.

The extinct family Dromomerycidae represents a distinct lineage of ruminants, further complicating any simple classification of pecoran cranial appendages. Dromomerycids, including *Dromomeryx* and *Cranioceras*, were endemic to North America from the late early Miocene to the early Pliocene and were probably related to Cervidae [80]. They possessed unusual, unbranched, nondeciduous appendages that superficially resembled elongated ossicones, together with additional posterior cranial appendages (Figure 10M). Because of these ossicone-like structures, dromomerycids were initially compared with giraffomorphs. However, other skeletal features indicate that they should not be included within this group. Their resemblance to giraffomorphs is therefore interpreted as a case of parallel or convergent evolution. Dromomerycids demonstrate that similar cranial appendage morphologies could evolve independently in distantly related pecoran lineages.

The extinct family Hoplitomerycidae represents another enigmatic lineage of Pecora, and its phylogenetic position remains controversial. Early studies placed hoplitomerycids close to the Cervidae [84]. However, other studies have rejected this close relationship and instead recovered Hoplitomerycidae as an early-diverging pecoran lineage that likely originated from a primitive lineage that split before the major radiation of the extant pecoran families [85]. Hoplitomerycids lived during the Early to Late Miocene, approximately 18–6 Mya, mainly on the east coast of Southern Italy. Most hoplitomerycids, including *Hoplitomeryx*, possessed five permanent osseous cranial appendages: a single median frontal appendage and two pairs of lateral appendages located above the orbits (Figure 10N). In contrast, *Scontromeryx* lacked the median frontal appendage and possessed only the paired supraorbital appendages [84]. These appendages consisted of solid bone and were not shed during life. Their external covering remains uncertain. The appendages are generally interpreted as exposed bony structures, resembling ossicones rather than the keratin-covered horns of bovids or the deciduous antlers of cervids. Nevertheless, because soft tissues are not preserved, the presence of a thin integument or skin covering cannot be excluded.

Another enigmatic extinct family of ruminants that deserves mention is Protoceratidae, whose phylogenetic position has long remained controversial. Traditionally, protoceratids were placed within Tylopoda and regarded as close relatives of camelids. However, recent studies support a closer affinity with Ruminantia [8]. Protoceratids were endemic to North America and first appeared during the middle Eocene (approximately 46–40 Mya), i.e., before the radiation of Pecora and the diversification of cranial appendages in extant pecoran families [86]. They were relatively common throughout the Eocene and Oligocene but became increasingly rare during the Miocene before becoming extinct in the early Pliocene. Although the earliest protoceratids lacked cranial appendages, males of more derived genera, including *Protoceras* and *Syndyoceras*, developed elongated cranial appendages that were sometimes distally branched (Figure 10O,P) [86,87]. These appendages lacked a keratinized sheath, and the numerous vascular grooves preserved on their surfaces indicate that they were covered by skin, resembling the ossicones of extant giraffids. Unlike any living pecoran lineage, protoceratids possessed a paired set of frontal appendages together with a single or paired median appendage in the nasal region, resulting in one of the most distinctive cranial appendage configurations known among Cenozoic artiodactyls. Because Protoceratidae represent an extinct side branch that diverged before the diversification of modern pecoran families, their cranial appendages are generally regarded as an independent evolutionary experiment rather than homologues of the horns, antlers, ossicones, or pronghorns of extant ruminants.

In summary, paleontological data do not yet resolve whether all pecoran cranial appendages derive from a single ancestral structure or whether they evolved independently in several lineages. The fossil record shows that the main types of cranial appendages appeared suddenly, or at least are first documented within a relatively short early Miocene interval, and that extinct forms displayed a much wider morphological range than extant ruminants. Moreover, several extinct ruminant families, including Dromomerycidae, Hoplitomerycidae, and Protoceratidae, evolved highly distinctive cranial appendages, yet their phylogenetic positions remain uncertain. This additional diversity further complicates attempts to reconstruct the evolutionary history and homology of ruminant cranial appendages. Bovids, cervids, antilocaprids, giraffomorphs, dromomerycids, hoplitomerycids, and protoceratids each show lineage-specific trajectories, while moschids remain hornless in the known fossil record. Therefore, paleontology supports a cautious interpretation: pecoran cranial appendages may share deep developmental predispositions associated with the frontal region of the skull, but the fossil record more clearly documents repeated, mosaic, and parallel evolution of diverse headgear morphologies than a simple transformation series from one appendage type into another.

## 7. Homology Versus Convergence of Ruminant Cranial Appendages: Evidence from Gene Expression and Comparative Genomics

As early as the mid-19th century, the French zoologist and anatomist Étienne Geoffroy Saint-Hilaire wondered whether horns and antlers are homologous or analogous organs [5,88]. He argued that this question should not be limited to just horns and antlers but should also consider unique structures like pronghorns and ossicones of giraffes and okapis. Fossil data do not make it easy to resolve this, as there was a great diversity of cranial appendages among extinct ruminants, some of them lacked horns and antlers, and the relationships among many extinct ruminants are unclear. A problematic example is the Miocene genus *Hoplitomeryx*. This animal had horns similar to those of bovids, but its skeleton resembled that of cervids [89]. This illustrates the remarkable evolutionary plasticity of ruminant cranial appendages.

Comparative transcriptomic analyses identified 624 genes specifically expressed in horn buds and 761 in antler buds, of which 201 were shared between both structures and enriched in pathways related to bone development, skin formation, and neurogenesis [90]. These findings further demonstrated that all pecoran cranial appendage types share a common structural organization, consisting of a frontal position, an osseous core, and a soft-tissue covering, while cranial appendage-specific genes show the strongest co-expression in bone, skin, neural tissues, and testes. Strong evidence that different types of ruminant cranial appendages share the same genetic basis comes from the fact that the *RXFP2* gene became non-functional (convergent pseudogenization) in two unrelated lineages that independently lost their cranial appendages: musk deer (Moschidae) and water deer (Hydropotinae). The loss of the same gene in phylogenetically distant lineages following secondary cranial appendage loss suggests the existence of a common molecular framework governing cranial appendage development. This interpretation is further supported by studies in domestic sheep, where mutations in *RXFP2* have been associated with hornlessness [90,91].

Direct transcriptomic evidence supporting a common evolutionary origin of horns and antlers was provided by comparative RNA-seq analyses of developing cranial appendages [92]. Young horn bud and antler tissues exhibited highly similar expression profiles and shared numerous homologous gene sets. Divergence between the two structures became apparent only at later developmental stages, suggesting that both originate from a conserved developmental program inherited from the common ancestor of Pecora, whereas their morphological differences arise through divergence during subsequent ontogenetic stages. Among the most informative shared genes, *ALX1*, a transcription factor involved in craniofacial neural crest morphogenesis, was highly expressed in both horn buds and antler pedicles, identifying it as a strong candidate ancestral regulator. Additional genes shared between the two structures included *WNT3*, *FGF10*, and *SOX9*. Furthermore, *TNRC6A*, *MYOC*, and *MAPK14* distinguished cranial appendage osteogenesis from osteogenesis elsewhere in the skull and may therefore represent components of the molecular identity of ruminant cranial appendages [92].

At the cellular level, the conservation of progenitor mesenchymal cell populations between goat horns and cervid antlers, the shared expression of *RXFP2* in antlerogenic periosteum-derived stem cells and horn bud osteoblasts, and the common origin of both horn and antler primordia from cranial neural crest cells (CNCCs) collectively support the hypothesis of a homologous developmental osseous core underlying ruminant cranial appendages [90,92,93,94,95]. According to this model, the diverse cranial appendage types of Pecora are proposed to derive from a single ancestral osseous cranial appendage present in their common ancestor, whereas the keratinous sheath of bovids, the deciduous antler of cervids, the branched sheath of pronghorns, and the ossicones of giraffids represent lineage-specific modifications of a shared developmental module. The common developmental framework of horns and antlers, rooted in the CNCC lineage and regulated by conserved factors such as *RXFP2*, *ALX1*, *SOX9*, and components of the Wnt signaling pathway, therefore provides strong support for their homologous evolutionary origin.

The keratin-based differentiation that distinguishes horns from antlers also appears to follow a partially shared genetic program. Several α-keratin type II genes, including *KRT82*, are expressed during horn development [9]. Notably, similar mutations affecting *KRT82* have been identified in both cattle and pronghorn, suggesting convergent evolution of the keratinous horn sheath in the Bovidae and Antilocapridae lineages. This observation highlights an important distinction between the evolutionary histories of different cranial appendage components: whereas the osseous core is likely homologous across Pecora, aspects of the keratinous covering may have arisen or diversified independently in different lineages.

## 8. Molecular and Cellular Mechanisms of Horn and Antler Development

Contemporary genomic, transcriptomic, proteomic, and single-cell analyses have substantially expanded our understanding of ruminant cranial appendage development. These studies show that horn and antler formation depends on the coordinated activity of specific progenitor cell populations, conserved developmental pathways, endocrine regulation, epithelial–mesenchymal interactions, immune-cell-mediated niche formation, and tightly regulated programs of chondrogenesis, osteogenesis, keratinization, angiogenesis, and tissue mineralization. The currently available evidence regarding developmental genes, signaling pathways, cell origins, and ossification mechanisms is summarized in Table 1.

A central finding from recent developmental studies shows that both horns and antlers derive from cranial neural crest cells (CNCCs). Genes associated with neural crest cell migration, including *SOX10*, *SNAI1*, *SNAI2*, *TFAP2A*, *NGFR*, and *COL11A2*, are specifically expressed in horn and antler primordial tissues, and the presence of SOX10 and NGFR proteins has been confirmed immunohistochemically in the embryonic horn bud of sheep [90]. This conclusion was further reinforced by RNA-seq transcriptomic analyses showing elevated expression of multiple CNCC markers, including *ALX1*, *CRABP1*, *DLX1*, *DLX2*, *SOX10*, *TFAP2A*, *TFAP2B*, and *TFAP2C*, in both developing horn buds of cattle and antler tissues of deer. This suggests that a CNCC-based embryonic origin may be conserved across ruminant cranial appendage types [92].

In bovine fetuses, the horn bud has been histologically characterized at 58 days of gestation, which represents the earliest developmental stage of horn formation examined to date in this species [93]. Compared with polled fetuses, horned fetuses exhibit a distinct horn bud structure characterized by epidermal thickening, a condensed cell layer beneath the epithelium, increased peripheral innervation, and epidermal localization of RXFP2 protein. These features indicate that horn initiation is not simply a local thickening of the skin but a spatially organized developmental process involving interactions among epithelial, neural, and mesenchymal components [93].

Transcriptomic studies support the view that horn initiation is controlled by a relatively small set of key regulatory genes rather than by extensive transcriptome-wide changes. The first transcriptomic analysis of the ovine embryonic horn bud showed that horn bud and frontal skin tissues share highly similar global expression profiles, with 21,425 genes expressed in both tissues. However, 68 differentially expressed genes, including 58 genes upregulated in the horn bud, distinguish the horn bud from surrounding skin [96]. These data indicate that horn development depends on the selective activation of specific regulatory programs within a broadly similar cranial skin environment.

Single-cell RNA sequencing has provided unprecedented resolution of the cell populations involved in horn and antler morphogenesis. In the bovine horn bud, keratinocytes, dermal cells, neural cells, and neural crest/chondrocyte progenitors have been identified. These data suggest that neural cells initiate horn bud development, CNCC-derived cells give rise to the osseous bony horn core, and keratinocytes form the keratinous sheath [97]. In the developing goat horn bud, single-cell RNA sequencing identified ZEB2-positive progenitor mesenchymal cells (ZEB2+ PMCs) as the key osteogenic population responsible for forming the osseous horn core [95]. These cells appear before ossification begins, express osteogenic genes including *RUNX2*, *SP7*, *ALPL*, *IBSP*, and *BGLAP*, and retain high osteogenic capacity both in vitro and in vivo. These findings are consistent with earlier reports linking deletions of *ZEB2*, which encodes Zinc finger E-box-binding homeobox 2, a transcription factor involved in the TGF-β signaling pathway, to polled phenotypes and developmental abnormalities in cattle [98]. *MEF2A* has also been identified as a co-regulator of *ZEB2* that activates osteogenic gene expression and is required for proper differentiation of ZEB2+ PMCs [95].

In antlers, development and regeneration depend on specialized stem and progenitor populations. A dedicated population of antler stem cells (ASCs) has been identified in the antlerogenic periosteum (AP), pedicle periosteum (PP), and reserve mesenchyme (RM) of the antler tip in elk (*Cervus canadensis*) [99]. The AP is a specialized periosteal tissue covering the frontal crest that initiates pedicle development and the formation of the first antler, whereas the PP covers the permanent pedicle and is essential for the annual regeneration of antlers after casting. ASCs residing in the AP, PP, and RM express classical mesenchymal stem cell markers, including *CD73*, *CD90*, *CD105*, *CD29*, *CD44*, *CD146*, and *Stro-1*, but lack the expression of canonical pluripotency markers such as *OCT4*, *SOX2*, and *NANOG*. They are also characterized by high expression of *c-MYC*, *TERT*, and the intermediate filament gene *Nestin*, consistent with their extraordinary proliferative capacity [99].

Further single-cell analysis of the antlerogenic periosteum in sika deer (*Cervus nippon*) identified a critical subpopulation of THY1+/RXFP2+ mesenchymal cells, referred to as RXFP2-positive antlerogenic periosteum mesenchymal cells (RXFP2+ APMCs). These cells give rise directly to antler progenitor cells (APPCs) and may be considered the “seed cells” that initiate postnatal antler development [94]. Like the broader antlerogenic periosteum, RXFP2+ APMCs express CNCC markers such as *TWIST1*, *PRRX2*, and *SNAI1*, and show markedly lower expression of HOX genes than periosteal cells of long bones. This expression pattern reflects their craniofacial neural crest origin and developmental plasticity [94].

Among the genes implicated in cranial appendage development, *RXFP2* occupies a central role in controlling horn and antler initiation. Convergent pseudogenization of *RXFP2* has been documented in two independently hornless pecoran lineages, Moschidae and Hydropotinae, while a 1.8 kb insertion in the 3′UTR of *RXFP2* causes the polled phenotype in sheep [90]. In Altay sheep, *RXFP2* was identified as the most significantly differentially expressed gene in the embryonic horn bud relative to frontal skin [96]. In cattle, RXFP2 protein is localized to the epidermis, peripheral nerves, and osteoblasts of the developing horn bud [93]. In deer, *RXFP2* has been recognized as a marker of antler stem cells in the antlerogenic periosteum [99] and later as a defining marker of the RXFP2+ APMC subpopulation involved in antler initiation [94]. *RXFP2* expression is also highly specific to cranial appendage tissues and is shared between horn buds and antler tissues [92]. Collectively, these findings establish *RXFP2* as a key regulator linking androgen signaling, progenitor-cell activation, and cranial appendage initiation across ruminant species.

**Figure 11:**
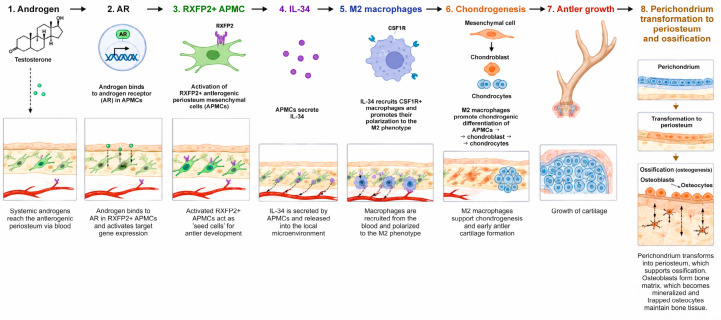
Molecular and cellular cascade underlying antler initiation, growth, and ossification. Androgen signaling activates androgen receptor (AR) in RXFP2-positive antlerogenic periosteum mesenchymal cells (APMCs), inducing their activation and the expression of pro-regenerative genes. Activated RXFP2+ APMCs secrete IL-34, which recruits and polarizes CSF1R-positive macrophages toward the M2 phenotype. M2 macrophages promote chondrogenic differentiation of mesenchymal progenitor cells, leading to the formation of chondroblasts and chondrocytes and subsequent rapid antler cartilage growth. During antler maturation, the perichondrium undergoes transformation into osteogenic periosteum, initiating endochondral ossification. Osteoblasts differentiate and produce bone matrix, while embedded osteoblasts mature into osteocytes, resulting in mineralized bone formation and antler elongation.

The Wnt/β-catenin pathway is a major downstream effector of horn and antler development. In antlerogenic periosteum, RXFP2+ APMCs maintain quiescence through expression of Wnt inhibitors, including *SFRP2*, *SFRP4*, *DKK2*, and *DKK3*, until the pubertal increase in androgen levels. The Wnt co-receptor LRP6 is enriched in the RXFP2+ subpopulation, and CRISPR-Cas9 knockout of *LRP6* significantly impairs the proliferative and osteogenic capacity of these cells [94]. These findings support a regulatory sequence in which androgen signaling activates the androgen receptor (AR), induces *RXFP2*, stimulates Wnt/β-catenin signaling, and promotes APMC proliferation (Figure 11) [94]. The involvement of Wnt signaling is also supported by transcriptomic data from the embryonic sheep horn bud, in which differentially expressed genes are enriched in the Wnt signaling pathway [96]. In sika deer antlers, *WNT11* is associated with the chondrogenic growth phase, whereas *WNT16* is upregulated during ossification [100]. A distinct mesenchymal stromal cell subpopulation, MSC_3, marked by *TNN* expression, which encodes tenascin-W, has been identified as a Wnt-mediated regulator that promotes chondrogenesis over osteogenesis during antler growth. Suppression of this subpopulation at the onset of ossification redirects mesenchymal cells toward the osteoblast lineage [101].

Epithelial-to-mesenchymal transition (EMT) and neural crest cell migration are also central to early horn bud formation and cranial appendage patterning. *HOXD1*, a member of the conserved *HOX* gene family, regulates neural crest cell migration and differentiation through modulation of Wnt signaling and EMT in mice [102]. Consistent with this role, *HOXD1* has been implicated in cranial appendage patterning, particularly in determining horn number and spatial arrangement in polycerate sheep and goats. A 4 bp deletion in *HOXD1* is associated with the polycerate phenotype in sheep, where it leads to splitting of the horn bud anlage [27]. In cattle, the Polled Celtic mutation (Pc) has been proposed to inhibit EMT and neural crest cell migration, resulting in reduced expression of genes involved in keratinization and cytoskeletal organization and thereby preventing horn bud development [97]. Further evidence for the involvement of neural crest patterning comes from the identification of a Pecora-specific highly conserved non-coding element (HCE), located 15 kb from the *HOXD* cluster and originating from a 3.6 kb transposon insertion. This element has been proposed as a regulator of cranial neural crest cell patterning and maps to the candidate genomic region associated with the polycerate phenotype in sheep [90].

The transition from progenitor activation to tissue growth involves tightly regulated programs of chondrogenesis, osteogenesis, keratinization, and extracellular matrix production. Osteogenesis in horn and antler tissues is regulated by a largely conserved set of transcription factors and extracellular matrix genes. In the goat horn bud, ZEB2+ PMCs show high expression of *RUNX2*, *BMP1*, *BMP2*, and *MEF2A*, while osteopontin, encoded by *SPP1* and secreted by osteogenic cells, promotes mineralization of the keratinous sheath [95]. In gene-edited cattle, elevated expression of keratin genes including *KRT4*, *KRT15*, *KRT17*, *KRT19*, and *KRT80* has been detected in the developing horn bud, highlighting the importance of keratinization in horn formation and structure [97]. Other keratin genes, including *KRT14*, *KRT36*, *KRT82*, and *KRT84*, are also elevated during horn development, whereas *DSG1*, encoding desmoglein 1, is enriched in horns relative to antlers. Desmoglein 1 is thought to strengthen epidermal intercellular adhesion and contribute to the exceptional mechanical durability of the bovid horn sheath [95].

In antlers, the chondrogenic growth phase is characterized by high expression of *SOX9*, *COL2A1*, *COL3A1*, *COL11A2*, *ACAN*, and *HAPLN1*, with *COL2A1* serving as a reliable marker of chondrogenesis initiation. In contrast, the ossification phase, during which bone tissue forms, is associated with increased expression of *S100*-family genes, *OPN*, and *OCN* [100]. Single-cell RNA sequencing of sika deer antlers at the growth stage, day 60, and ossification stage, day 90, identified eight major cell types: mesenchymal cells, chondrocytes, osteoblasts, pericytes, endothelial cells, monocytes/macrophages, osteoclasts, and NK cells. The proportions of these cell types changed markedly during development; in particular, osteoblasts increased from 5.4% to 29.8%, and osteoclasts increased from 1.6% to 10.9% during ossification [101]. Independent single-cell RNA sequencing analyses further demonstrated extensive proliferation of progenitor cells, endothelial cells, and mural cells, which are vessel-associated support cells surrounding blood vessels, during antler development [103]. This is consistent with the exceptionally rapid growth rate of antlers, which requires a continuous and abundant supply of oxygen and nutrients. Robust angiogenesis and vascular expansion are therefore essential to sustain the intense metabolic demands of antler growth. In this study, antler progenitor cells were classified into five subclusters. Notably, subcluster 4 of cells showed high expression of AR, indicating that this population may serve as the primary responder to androgen signaling. Because antler development is triggered by rising androgen levels, these SC4 cells are proposed to play a key role in initiating antler growth [103].

Endocrine regulation, particularly androgen signaling, is central to antler initiation and ossification. The pubertal increase in androgen levels activates AR in RXFP2+ APMCs, leading to their activation and induction of pro-regenerative genes. During antler ossification, upregulation of *AR*, *CYP17A1*, *CYP19A1*, *SHBG*, and *SRD5A1* suggests that testosterone and its metabolites, including dihydrotestosterone (DHT) and estrogens, act as principal regulators of antler mineralization [100]. These data link systemic endocrine signals with local progenitor-cell activation, chondrogenic growth, and subsequent mineralization.

Recent work has also revealed that antler development is regulated by an immunological niche (Figure 11). RXFP2+ APMCs actively shape their own developmental environment by secreting IL-34, which recruits CSF1R-positive macrophages and promotes their polarization toward the M2 phenotype [36,94]. These M2 macrophages stimulate the chondrogenic differentiation of antlerogenic mesenchymal cells and are required for postnatal antler initiation. This finding identifies macrophages as key regulators of an androgen-dependent regenerative process and reveals an unexpected immune component of antler development [36,94].

Intercellular communication during annual antler regeneration is further mediated by extracellular vesicles (EVs) released by antler stem cells [104]. These EVs transport a diverse repertoire of growth factors, including IGF-1, VEGF, FGF, TGF-β1, and Galectin-1, together with regulatory microRNAs such as miR-21-5p, miR-143-3p, miR-140-3p, and miR-199a-3p. Through modulation of the Wnt/β-catenin, TGF-β, NF-κB, and STAT3 signaling pathways, these extracellular vesicles coordinate proliferation, angiogenesis, chondrogenesis, and osteogenesis [104].

Additional analyses show that variation in antler growth rate is associated with differential expression of *FBP2*, *TPT1*, *TFRC*, *PHOSPHO1*, *ZEB1*, and *NFATC2*. These expression patterns are linked to regulation through the PI3K–Akt, TGF-β, MAPK, and Wnt signaling pathways, with further coordination provided by microRNAs including miR-140, let-7e, and miR-296-3p [105]. This indicates that antler growth rate is not controlled by a single pathway but by an integrated regulatory network coordinating metabolism, proliferation, vascularization, chondrogenesis, and ossification.

The molecular and cellular processes described above are reflected in the spatial organization of the growing antler. Proteomic analysis revealed marked regional specialization: the antler tip, which functions as the growth center, is characterized by the predominance of protein synthesis machinery and molecular chaperones, together with the highest concentrations of IGF-1 and IFN-γ. In contrast, the mid-section, corresponding to the mineralizing zone, shows elevated levels of vascular proteins and alkaline phosphatase activity essential for calcium incorporation [106].

Several additional genes have also been implicated in cranial appendage development. *FOXL2* participates in horn development and is strongly associated with polled intersex syndrome (PIS) in goats through interactions with *SOX9* [107]. *TWIST1*, another regulator interacting with *SOX9*, has been linked to the development of scurs, or rudimentary horns [108]. Together with *RXFP2*, *HOXD1*, *ZEB2*, *MEF2A*, *SOX9*, Wnt pathway components, keratin genes, and extracellular matrix regulators, these genes form part of a broader developmental network that links cranial neural crest-derived progenitors, local tissue patterning, endocrine activation, immune-cell-mediated niche formation, and skeletal and epidermal differentiation.

## 9. The Antler as a Model of Controlled Oncogenesis: Proto-Oncogenes and Tumor Suppressors

A unique feature of antlers is their extraordinarily rapid growth rate of approximately 2 cm per day, which even exceeds the proliferation rate of malignant tumors [109]. Transcriptomic analyses have revealed that this remarkable growth relies on the activation of proto-oncogenic pathways while simultaneously maintaining effective anti-tumor control mechanisms. Substantial similarities have been identified between the molecular regulation of antler growth and osteosarcoma, leading to the hypothesis that genetic programs normally associated with tumor-like bone proliferation were evolutionarily co-opted for antler development [90]. This process is associated with the activation of growth-promoting genes that support intense cell proliferation within the cartilage of the growing antler [103]. The strong sexual selection acting on antler traits is thought to have maintained and elaborated these genetic programs, contributing to the predominantly male expression of antlers in most cervid species, with reindeer representing a notable exception.

Several proto-oncogenes, including *FOS*, *FAM83A*, and *REL*, were identified as targets of positive selection in the cervid lineage [90]. Among these, *FOS* is a well-established regulator of cell proliferation and differentiation, and its overexpression can induce osteosarcoma in mice through transformation of chondroblasts and osteoblasts. Antler-specific expression of *FGF19*, *FGF21*, *FGFBP3*, *PDGFD*, and *PDGFRL*, growth factors frequently associated with tumor cell proliferation, has also been reported [90]. Further evidence for the involvement of tumor-related pathways comes from the identification of 11 oncogenes (including *MYCN*, *GLI1*, *WNT3*, and *LMO1*) and 25 tumor suppressor genes (including *GADD45G*, *FAS*, *DNMT1*, and *WNT11*) whose expression changes during antler growth [100]. Despite exhibiting proliferation rates comparable to those observed in rapidly growing tumors, antlers maintain strict control over cell growth through the coordinated activity of oncogenes and tumor suppressor genes, thereby preventing neoplastic transformation [100].

Zoo-based comparative datasets have suggested that cervids may show a lower recorded cancer incidence than many other mammalian groups, with reported values of approximately 0.4–0.8% in cervids compared with 2.1–4.6% across broader zoo mammal datasets [90]. Zoo records are affected by uneven taxonomic sampling, diagnostic intensity, necropsy frequency, and small sample sizes for many species. Therefore, these data should not be taken as definitive evidence that cervids are generally cancer-resistant. However, these findings encourage further investigation into why cervids are tumor-resistant.

*PML*, which encodes a transcriptional co-activator of p53, exhibited the strongest signal of positive selection in the cervid lineage, with accumulation of 11 lineage-specific amino acid substitutions. In addition, three p53 co-factors (*PML*, *NMT2*, and *CD2AP*) and five p53 regulators (*ELOVL6*, *S100A8*, *ISG15*, *CNOT3*, and *CCDC69*) showed evidence of positive selection, suggesting that the TP53 tumor-suppressor pathway has been strengthened during evolution [90]. This mechanism differs from that observed in elephants, where cancer resistance is associated with amplification of *TP53* copy number, indicating two independent evolutionary strategies acting on the same anti-cancer pathway. Additional anti-tumor genes under positive selection in cervids include the extracellular matrix regulator *ADAMTS18* and the DNA damage response genes *SLF1*, *RHNO1*, *DDB2*, *TP73*, and *TP53I13*, of which *TP73* and *TP53I13* are specifically expressed in antler tissue [90].

Evidence for anti-tumor activity has also been obtained at the protein level. Extracts obtained from the tip of growing antlers exhibited anti-tumor effects both in vitro and in vivo against glioblastoma, prostate, colon, and breast cancer cell lines, whereas this activity was absent from the mineralizing mid-section of the antler [106]. These observations suggest that the rapidly growing antler tip contains bioactive molecules capable of modulating proliferation-related pathways.

Several molecular mechanisms have been proposed to explain how antler tissues maintain rapid growth while avoiding malignant transformation. High expression of *c-MYC* has been detected in antler stem cells, where it appears to contribute to the balance between rapid proliferation and oncogenic control [99]. During ossification, increased expression of the ribosomal proteins RPL37, RPL23, RPS20, and RPL26 enhances the stability and activity of p53, thereby promoting cell-cycle arrest and apoptosis in antler cells. This mechanism likely contributes to the termination of the rapid antler growth phase [101].

The analogy between antler growth and oncogenesis has several important limitations. Antlerogenesis is a normal, cyclic, and spatially organized developmental process, whereas cancer is characterized by uncontrolled growth, genetic instability, invasion, and loss of tissue organization. Despite the promising findings, the translational medical significance remains uncertain because the available studies rely primarily on crude tissue extracts, limited experimental models, heterogeneous antler tissues, and lack clinical validation. At present, these observations are best interpreted as evidence that growing antler tissue contains bioactive molecules capable of modulating proliferation-related pathways rather than as evidence for direct therapeutic potential.

## 10. Conclusions

Ruminant cranial appendages display remarkable structural diversity, ranging from permanent horns and annually shed antlers to pronghorns and ossicones. These structures differ substantially in anatomy, developmental mechanisms, growth patterns, persistence, covering tissues, and function. These similarities and differences are summarized in Table 1. Comparative developmental data, especially the contrast between dermally ossifying bovid horn cores and cervid antlers arising from frontal bone pedicles, support the possibility that some appendage types evolved independently or underwent extensive lineage-specific reorganization. Paleontological evidence also remains inconclusive, because the main appendage types appear in the fossil record within a relatively short early Miocene interval and no clearly transitional common ancestral form has yet been identified.

Conversely, several features are consistent with the common-origin hypothesis: ruminant cranial appendages are paired structures associated with the frontal region of the skull, they include an osseous component, and recent genomic, transcriptomic, and single-cell analyses reveal shared developmental pathways, CNCC-derived progenitor signatures, and conserved regulatory genes such as *RXFP2*, *ALX1*, *SOX9*, and components of Wnt signaling. These data suggest that horns, pronghorns, antlers, and ossicones may share a deep developmental module, even if their external morphology and growth patterns evolved in a lineage-specific manner.

Therefore, the question of whether pecoran cranial appendages are homologous structures inherited from a common ancestor or analogous structures produced by convergent evolution remains unresolved. Current evidence supports a cautious intermediate interpretation: the four appendage types may not be homologous in all of their components, but they may share an ancestral developmental capacity of the cranial neural crest-derived frontal region. Resolving this issue will require new fossil discoveries, especially late Oligocene or earliest Miocene stem pecorans preserving incipient paired frontal appendages, together with comparative developmental and molecular studies of all four appendage types, particularly pronghorns and ossicones.

## Figures and Tables

**Table 1:** Comparative developmental and molecular characteristics of the four types of ruminant cranial appendages.

Appendages	Horns	Pronghorns	Antlers	Ossicones
**Family**	Bovidae	Antilocapridae	Cervidae	Giraffidae
**Adult covering**	Permanent keratin sheath	Deciduous keratin sheath	Velvet during growth; shed before rut	Skin with hair
**Persistence**	Permanent	Permanent osseous core; keratin sheath shed annually	Antler shed and regenerated annually; permanent pedicle	Permanent
**Growth pattern**	Basal growth	Basal growth	Growth at tip	Surface (peripheral) growth
**Ossification mode**	Intramembranous ossification	Uncertain	Endochondral ossification	Controversial (intramembranous vs. endochondral)
**Cartilage precursor**	Absent	Unknown	Present	Controversial
**Fusion with frontal bone**	Secondary fusion after birth	Present	Pedicle continuous with frontal bone	Secondary fusion after ossification
**Regenerative capacity**	No	No; only keratin sheath regenerated annually	Complete annual regeneration	No
**Stem-cell niche**	Putative stem/progenitor niche	Unknown	Pedicle periosteum and reserve mesenchyme	Unknown
**Cranial neural crest origin**	Supported	Presumed	Supported	Presumed
**Major developmental signaling pathways**	Wnt/β-catenin, BMP, FGF, Hedgehog, androgen signaling	No molecular studies available	Wnt/β-catenin, BMP, FGF, Hedgehog, androgen signaling	No molecular studies available
**Key regulatory genes**	*RXFP2*, *ALX1*, *SOX9*, *RUNX2*, *TWIST1*, *HOXD1*, *ZEB2*, *MEF2A*	No molecular studies available	*RXFP2*, *ALX1*, *SOX9*, *RUNX2*, *SP7*, *SNAI1/2*, *TWIST1*, multiple stem-cell markers	No molecular studies available
**Role of sex hormones**	Strong androgen dependence	Probably androgen dependent	Androgens regulate regeneration and antler cycle	Probably androgen dependent
**Major unresolved questions**	Identity of horn stem cells; mechanisms restricting regeneration	Cellular origin, ossification mode, molecular regulation	Mechanisms enabling complete epimorphic regeneration	Ossification mechanism, embryonic origin, molecular regulation

Bold text indicates column headings and the characteristics compared.

## Data Availability

No new data were created or analyzed in this study. Data sharing is not applicable to this article.

## Abbreviations

The following abbreviations are used in this manuscript:

ACAN	aggrecan
ADAMTS18	ADAM metallopeptidase with thrombospondin type 1 motif 18
ALPL	alkaline phosphatase
ALX1	ALX homeobox 1
AP	antlerogenic periosteum
APMCs	antlerogenic periosteum mesenchymal cells
APPCs	antler progenitor cells
AR	androgen receptor
ASCs	antler stem cells
BGLAP	bone gamma-carboxyglutamate protein; osteocalcin
BMP1	bone morphogenetic protein 1
BMP2	bone morphogenetic protein 2
CCDC69	coiled-coil domain containing 69
CCND1	cyclin D1
CD2AP	CD2-associated protein
CD29	cluster of differentiation 29; integrin β1/ITGB1
CD44	cluster of differentiation 44
CD73	cluster of differentiation 73; ecto-5′-nucleotidase/NT5E
CD90	cluster of differentiation 90; THY1
CD105	cluster of differentiation 105; endoglin/ENG
CD146	cluster of differentiation 146; MCAM
c-MYC	MYC proto-oncogene
CNCCs	cranial neural crest cells
CNOT3	CCR4-NOT transcription complex subunit 3
COL2A1	collagen type II alpha 1 chain
COL3A1	collagen type III alpha 1 chain
COL11A2	collagen type XI alpha 2 chain
CRABP1	cellular retinoic acid binding protein 1
CSF1R	colony stimulating factor 1 receptor
CYP17A1	cytochrome P450 family 17 subfamily A member 1
CYP19A1	cytochrome P450 family 19 subfamily A member 1; aromatase
DDB2	damage specific DNA binding protein 2
DHT	dihydrotestosterone
DKK2	dickkopf WNT signaling pathway inhibitor 2
DKK3	dickkopf WNT signaling pathway inhibitor 3
DLX1	distal-less homeobox 1
DLX2	distal-less homeobox 2
DNMT1	DNA methyltransferase 1
DSG1	desmoglein 1
ELOVL6	elongation of very long chain fatty acids protein 6
FAM83A	family with sequence similarity 83 member A
FAS	Fas cell surface death receptor
FBP2	fructose-bisphosphatase 2
FGF	fibroblast growth factor
FGF10	fibroblast growth factor 10
FGF19	fibroblast growth factor 19
FGF21	fibroblast growth factor 21
FGFBP3	fibroblast growth factor binding protein 3
FOS	Fos proto-oncogene, AP-1 transcription factor subunit
FOXL2	forkhead box L2
GADD45G	growth arrest and DNA damage inducible gamma
GLI1	GLI family zinc finger 1
HAPLN1	hyaluronan and proteoglycan link protein 1
HCE	highly conserved non-coding element
HOX	homeobox gene family
HOXD	homeobox D gene cluster
HOXD1	homeobox D1
IBSP	integrin binding sialoprotein
IFN-γ	interferon gamma
IGF-1	insulin-like growth factor 1
IL-34	interleukin 34
ISG15	ISG15 ubiquitin-like modifier
KRT4	keratin 4
KRT14	keratin 14
KRT15	keratin 15
KRT17	keratin 17
KRT19	keratin 19
KRT36	keratin 36
KRT80	keratin 80
KRT82	keratin 82
KRT84	keratin 84
let-7e	microRNA let-7e
LMO1	LIM domain only 1
LRP6	LDL receptor related protein 6
MAPK	mitogen-activated protein kinase
MAPK14	mitogen-activated protein kinase 14
MEF2A	myocyte enhancer factor 2A
miR-21-5p	microRNA-21-5p
miR-140	microRNA-140
miR-140-3p	microRNA-140-3p
miR-143-3p	microRNA-143-3p
miR-199a-3p	microRNA-199a-3p
miR-296-3p	microRNA-296-3p
MYCN	MYCN proto-oncogene
MYOC	myocilin
NANOG	Nanog homeobox
NF-κB	nuclear factor kappa-light-chain-enhancer of activated B cells
NFATC2	nuclear factor of activated T cells 2
NGFR	nerve growth factor receptor
NK	natural killer cells
NMT2	*N*-myristoyltransferase 2
OCN	osteocalcin
OCT4	octamer-binding transcription factor 4
OPN	osteopontin
PDGFD	platelet-derived growth factor D
PDGFRL	platelet-derived growth factor receptor-like
PHOSPHO1	phosphoethanolamine/phosphocholine phosphatase 1
PI3K–Akt	phosphoinositide 3-kinase–Akt pathway
PIS	polled intersex syndrome
PML	promyelocytic leukemia protein
PMCs	progenitor mesenchymal cells
PP	pedicle periosteum
PRRX2	paired related homeobox 2
REL	REL proto-oncogene, NF-κB subunit
RHNO1	RAD9-HUS1-RAD1 interacting nuclear orphan 1
RM	reserve mesenchyme
RPL23	ribosomal protein L23
RPL26	ribosomal protein L26
RPL37	ribosomal protein L37
RPS20	ribosomal protein S20
RUNX2	runt-related transcription factor 2
RXFP2	relaxin/insulin-like family peptide receptor 2
S100A8	S100 calcium-binding protein A8
SFRP2	secreted frizzled-related protein 2
SFRP4	secreted frizzled-related protein 4
SHBG	sex hormone-binding globulin
SLF1	SMC5-SMC6 complex localization factor 1
SNAI1	snail family transcriptional repressor 1
SNAI2	snail family transcriptional repressor 2
SOX2	SRY-box transcription factor 2
SOX9	SRY-box transcription factor 9
SOX10	SRY-box transcription factor 10
SP7	Sp7 transcription factor; osterix
SPP1	secreted phosphoprotein 1; osteopontin
SRD5A1	steroid 5 alpha-reductase 1
STAT3	signal transducer and activator of transcription 3
Stro-1	a protein marker of mesenchymal stem cells (MSC)
TERT	telomerase reverse transcriptase
TFAP2A	transcription factor AP-2 alpha
TFAP2B	transcription factor AP-2 beta
TFAP2C	transcription factor AP-2 gamma
TFRC	transferrin receptor
TGF-β	transforming growth factor beta
TGF-β1	transforming growth factor beta 1
THY1	Thy-1 cell surface antigen
TNN	tenascin N
TNRC6A	trinucleotide repeat containing adaptor 6A
TP53	tumor protein p53
TP53I13	tumor protein p53 inducible protein 13
TP73	tumor protein p73
TPT1	tumor protein, translationally controlled 1
TWIST1	twist family bHLH transcription factor 1
VEGF	vascular endothelial growth factor
Wnt	Wingless/Int-1-related signaling pathway
WNT3	Wnt family member 3
WNT11	Wnt family member 11
WNT16	Wnt family member 16
ZEB1/2	zinc finger E-box-binding homeobox 1/2
ZEB2+ PMC	ZEB2-positive progenitor mesenchymal cells

## Appendix A

Sources and copyright/license information for images used in the figures.

**Figure 3**. (A) Photograph ID 103009582 © Olexandr Taranukhin, Dreamstime.com. (B) Photograph by Dennis G. Jarvis, Wikimedia Commons, CC BY-SA 2.0. (C) Photograph by Michael Barera, Wikimedia Commons, CC BY-SA 4.0. (D–F) Reproduced from Aguirre et al. [110], CC BY 4.0. (G) Photograph by Chris Dodds, via Wikimedia Commons, CC BY-SA 2.0. (H) Photograph by John Cummings, via Wikimedia Commons, CC BY-SA 3.0.

**Figure 5**. (A) Photograph by Tobias Klenze, via Wikimedia Commons, CC BY-SA 4.0. (B) Photograph courtesy of Victor S. Cox and Thomas F. Fletcher, University of Minnesota Veterinary Anatomy Museum. Reproduced with permission.

**Figure 6**. (A) Photograph by Jaime E. Jiménez, via Wikimedia Commons, CC BY-SA 3.0. (B) Photograph by Mongo, via Wikimedia Commons, public domain. (C) Photograph by Schlaier, via Wikimedia Commons, CC BY 3.0. (D) Photograph by Walter Baxter, via Geograph/Wikimedia Commons, CC BY-SA 2.0. (E) Photograph by Rafal Piprek. (F) Photograph by sarefo, via Wikimedia Commons, CC BY-SA 3.0. (G) Photograph by Ryan Somma, via Wikimedia Commons, CC BY-SA 2.0. (H) Photograph by Paweł Kowalczyk. Reproduced with permission. (I) Photograph by Jazzix, via Wikimedia Commons, CC BY-SA 4.0. (J) Photograph by Staffan Widstrand, courtesy of bioGraphic. Reproduced with permission.

**Figure 8**. (A) Photograph by Bernard Dupont, via Wikimedia Commons, CC BY-SA 2.0. (B) Photograph by Greg Goebel, via Wikimedia Commons, CC BY 2.0. (C) Photograph by Klaus Rassinger and Gerhard Cammerer (Museum Wiesbaden), via Wikimedia Commons, CC BY-SA 3.0. (D) Adapted from Danowitz et al. [111], CC BY 4.0.

**Figure 10**. (A) Photograph by Ghedoghedo, via Wikimedia Commons, CC BY-SA 4.0. (B) Illustration by Caz41985, via Wikimedia Commons, CC BY-SA 4.0. (C) Illustration by R. Bruce Horsfall, from Scott [112], public domain. (D) Photography from the American Museum of Natural History, public domain. (E) Photograph by Ghedoghedo, via Wikimedia Commons, CC BY-SA 3.0. (F) Photograph by Jonathan Chen, via Wikimedia Commons, CC BY-SA 4.0. (G) Photograph by Didier Descouens, via Wikimedia Commons, CC BY-SA 4.0. (H) Photograph by Ghedoghedo, via Wikimedia Commons, public domain. (I) Photograph by Jonathan Chen, via Wikimedia Commons, CC BY-SA 4.0. (J) Photograph by Didier Descouens, via Wikimedia Commons, CC BY-SA 4.0. (K) Illustration by Nobu Tamura, via Wikimedia Commons, CC BY-SA 4.0. (L) Photograph by Susan B. Hochgraf, via Wikimedia Commons, CC0 1.0. (M) Photograph by FunkMonk, via Wikimedia Commons, CC BY-SA 3.0. (N) Photograph by Ghedoghedo, via Wikimedia Commons, CC BY-SA 3.0. (O) Photograph by Ryan Somma, via Wikimedia Commons, CC BY-SA 2.0. (P) Illustration from Osborn [113], via Wikimedia Commons, public domain.
